# Identification of aceNKPs, a committed common progenitor population of the ILC1 and NK cell continuum

**DOI:** 10.1073/pnas.2203454119

**Published:** 2022-11-29

**Authors:** Noe Rodriguez-Rodriguez, Paula A. Clark, Mayuri Gogoi, Ana C. F. Ferreira, Bernhard Kerscher, Alastair Crisp, Helen E. Jolin, Jane E. Murphy, Meera Sivasubramaniam, Luisa Pedro, Jennifer A. Walker, Morgan W. D. Heycock, Jacqueline D. Shields, Jillian L. Barlow, Andrew N. J. McKenzie

**Affiliations:** a Medical Research Council, Laboratory of Molecular Biology, Cambridge, CB2 0QH, United Kingdom; b Paul-Ehrlich-Institute, Federal Institute for Vaccines and Biomedicines, Langen 63225, Germany; c Hutchison/MRC Research Centre, Cambridge, CB2 0XZ, United Kingdom

**Keywords:** NK cells, ILC1s, innate lymphoidcells, NKG2A/C/E, hematopoiesis

## Abstract

The development of innate lymphoid cell (ILC) transcription factor reporter mice has shown a previously unexpected complexity in ILC hematopoiesis. Using novel polychromic mice to achieve higher phenotypic resolution, we have characterized bone marrow progenitors that are committed to the group 1 ILC lineage. These common ILC1/NK cell progenitors (ILC1/NKP), which we call “aceNKPs”, are defined as lineage^−^Id2^+^IL-7Rα^+^CD25^−^α4β7^−^NKG2A/C/E^+^Bcl11b^−^. In vitro, aceNKPs differentiate into group 1 ILCs, including NK-like cells that express Eomes without the requirement for IL-15, and produce IFN-γ and perforin upon IL-15 stimulation. Following reconstitution of *Rag2^−/−^Il2rg^−/−^* hosts, aceNKPs give rise to a spectrum of mature ILC1/NK cells (regardless of their tissue location) that cannot be clearly segregated into the traditional ILC1 and NK subsets, suggesting that group 1 ILCs constitute a dynamic continuum of ILCs that can develop from a common progenitor. In addition, aceNKP-derived ILC1/NK cells effectively ameliorate tumor burden in a model of lung metastasis, where they acquired a cytotoxic NK cell phenotype. Our results identify the primary ILC1/NK progenitor that lacks ILC2 or ILC3 potential and is strictly committed to ILC1/NK cell production irrespective of tissue homing.

Natural killer (NK) cells play key roles in immune responses against infections and tumors (1, 2). Since their identification in the 1970s, their ontogeny and physiological functions have been studied extensively (3–6). However, in the last decade, the discovery of new members of the innate lymphoid cell (ILC) family has revealed that the ontogeny and physiology of innate lymphocytes is more complex and richer than previously anticipated (7–11). Although closely related and codependent on the transcription factor T-bet for their development and IFN-γ production, ILC1 and NK cells have been reported to differ in their cytolytic activity with NK cells displaying Eomesodermin and perforin expression and superior granzyme production (12–14). By contrast, ILC2 development is regulated by high Gata3, Bcl11b, and RORα expression resulting in the expression of type-2 cytokines such as IL-5 and IL-13 (15–18), while IL-17A and IL-22-producing ILC3 require the induction of RORγt for their differentiation (19, 20).

The existing paradigm of NK cell ontogeny specifies that mature and terminally differentiated cytotoxic NK cells develop from bone marrow NK cell progenitors (NKPs), named pre-NKPs and refined (r) NKPs, after transitioning through an immature NK cell (iNK) state, further subdivided into several developmental phases (3, 6, 21–24). Early studies concluded that common lymphoid progenitors (CLPs) mature into Id2-positive intermediate ILC progenitor states with progressively more restricted lineage choices, with NKPs segregating from those precursors directed toward the development of ILC1, ILC2, and ILC3 (11, 25, 26). However, several recent reports have indicated that previously identified ILC progenitors retained an unrecognized capacity to produce NK cells, suggesting greater complexity to the differentiation and commitment process (27–30). Indeed, the advances in combinatorial transcription factor reporter mice used concurrently with cell surface markers have started to add even greater refinement to the models for ILC and NK cell development and lineage commitment, allowing both in vitro and in vivo analyses (15, 27, 28, 30, 31).

The analysis of ILC/NK development in 5× polychromILC mice identified a bifurcation of ILC2Ps from a mixed population of ILC1/NK/ILC3 progenitors (27), though the lineage restriction of the ILC1/NK/ILC3 progenitors was not tested. Prior studies using cell surface markers have suggested that the expression of NKG2D on CD122^+^ iNKs represents the first commitment step of NK cells from a T cell/pre-NK progenitor, correlating with the expression of the transcription factor inhibitor of DNA binding 2 (Id2) (21, 23, 32, 33), which binds to E proteins to preclude T cell development (33, 34). This developmental step has also been shown to require Nfil3/E4bp4 (35–38) and Tox (39). However, with the discovery of other ILC populations, it has become apparent that, Nfil3, Id2, and Tox are common to ILC and NKPs (37, 40–42), raising the question as to whether the existing model/markers for early NK cell commitment excludes additional ILC lineage potential. Also, critical for NK cell development is the expression of the related T-box binding transcription factors Eomesodermin (*Eomes*) and T-bet (*Tbx21*) (14, 43, 44). Mice that lack Eomes fail to develop NK cells, while T-bet-deficient mice continue to produce normal numbers of naïve NK cells that are incapable of terminal differentiation to an activated cytotoxic phenotype (5, 44–47). Although this indicates a functionally nonredundant role for Eomes, it is also evident that T-bet can play a reciprocal role in NK cell development in peripheral tissues such as the liver.

To address the study of ILCP commitment toward a restricted ILC1/NK cell lineage precursor, we have used previously existing and novel multiallele transcription factor reporter mice, cell surface phenotyping, single-cell RNA sequencing, and both in vitro and in vivo analyses of purified progenitor populations to provide high-resolution tracking of early ILC1/NK cell commitment from ILCPs. Using this combinatorial approach, we have been able to reveal Lin^−^Id2^+^IL-7Rα^+^CD25^−^α4β7^−^NKG2A/C/E^+^Bcl11b^−^ NKPs committed solely to the ILC1/NK lineage independent of tissue residence that are able to provide robust cytotoxicity in a mouse model of lung metastasis. We termed these progenitors ‘aceNKPs’.

## Results

### Expression of NKG2A/C/E in Transitional States of BM ILCPs Defines a Population of Progenitors Resembling NKPs

**Fig. 1:**
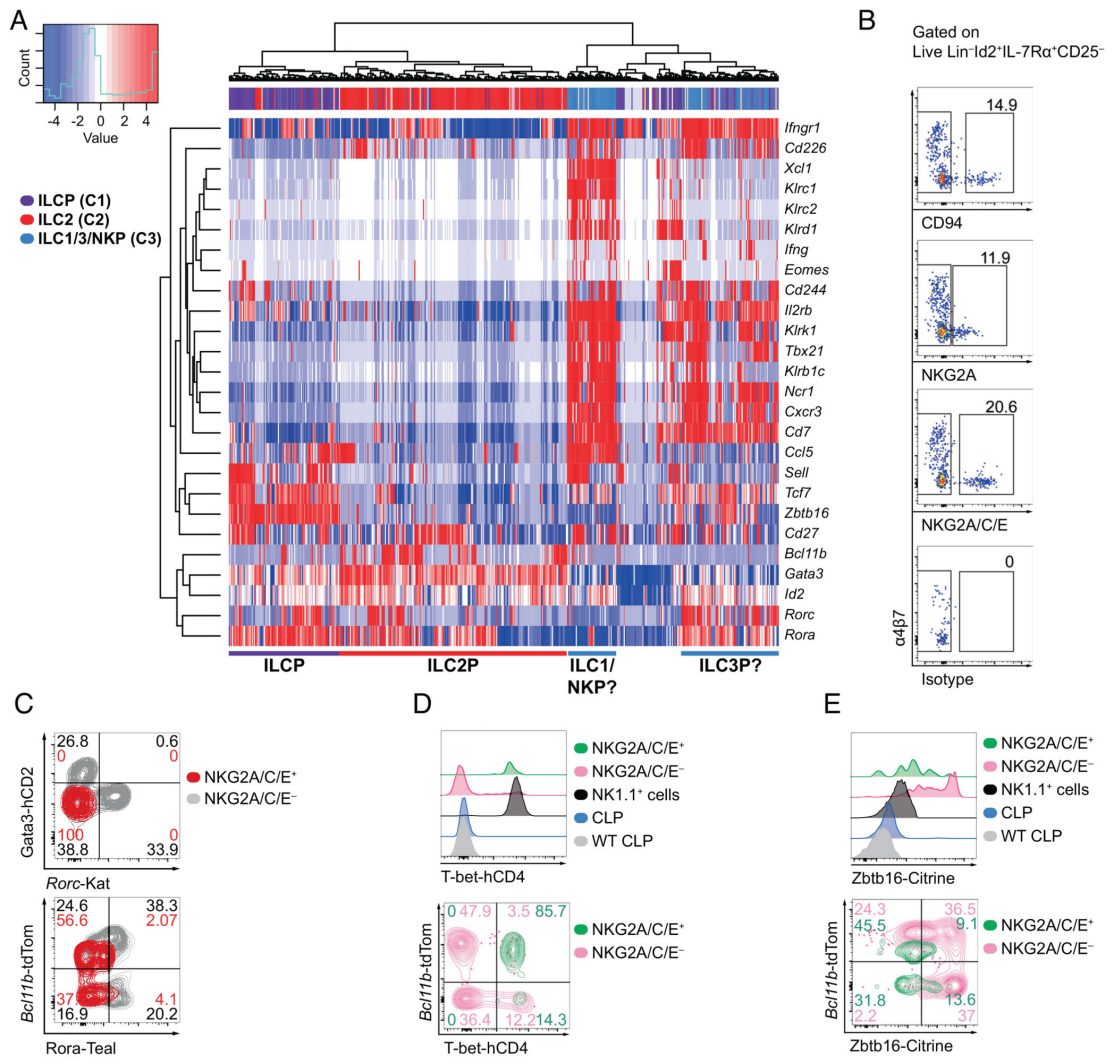
Expression of CD94 and NKG2A/C/E defines a subcluster of hematopoietic progenitors in the bone marrow that resemble type-1 ILC progenitors. (*A*) Expression of genes characterizing a group of putative ILC1, ILC3, and NK progenitors detected in the bone marrow of 5× polychromILC reporter mice by single-cell RNA sequencing from our previously published dataset GSE131038. ILCP (C1, purple), ILC2P (C2, red), and ILC1/3/NKP (C3, blue). (*B*) Bone marrow extracted from the hind limb bones (femora, tibiae, and ilia) of 5× polychromILC reporter mice was analyzed for the expression of CD94 (*Klrd1*), NKG2A (*Klrc1*), and NKG2A/C/E (*Klrc1/2/3*), within Lin^−^Id2^+^IL-7Rα^+^CD25^−^ cells. (*C*) Expression of RORα-Teal, *Rorc*-Katushka, *Bcl11b*-tdTomato and Gata3-hCD2 in NKG2A/C/E-positive and -negative cells from (*B*). Concatenated dot/contour plots from three samples are presented. Results are representative of two experiments (n = 6). (*D*) Expression of T-bet-hCD4 from 6× polychromILC reporter mice (*Id2*^+/BFP^, *Gata3*^+/hCD2^, *Rora*^+/Teal^, *Bcl11b*^+/tdTomato^, *Rorc*^+/Katushka^, *Tbx21*^+/hCD4^). Plots are representative of one out of two independent experiments (n = 11–12). (*E*) Expression of Zbtb16-Citrine from 5× polychromILC mice (*Id2*^+/BFP^, *Gata3*^+/hCD2^, *Bcl11b*^+/tdTomato^, *Rorc*^+/Katushka^, *Zbtb16*^+/Citrine^). Numbers depicted in panels indicate frequency of cells. Plots are representative of one out of two independent experiments (n = 11–12).

After the discovery of ILCs (8, 39, 48, 49), the identification and phenotypic characterization of bone marrow NK cell-committed progenitors (21, 23) has been challenged by later reports suggesting that pre-NKPs/rNKPs are a mixed population containing progenitors for ILC1 and ILC2s in addition to NK cells (50). Therefore, we aimed to isolate and characterize a uniquely NK cell-committed progenitor taking advantage of our analysis of single-cell-RNA sequencing (scRNAseq) data from highly resolved ILCPs using our previously generated polychromILC mice (15, 27). scRNAseq data and pseudotime analysis of bone marrow (BM) ILCPs using these multiple (5×) transcription factor reporter mouse strains indicated the presence of previously unappreciated transitional ILC precursor (transILCP) states downstream of ILCPs (27). Pseudotime analysis indicated that these transILCPs followed a trajectory toward a division of the ILC2Ps from the precursors of ILC1, ILC3, and NK cells. Notably, while the heterogeneous mixture of these predicted ILC1/3/NKPs was markedly different from ILCPs and ILC2Ps, these *Ncr1*^+^, *Cxcr3*^+^, *Cd7*^+^ ILC1/3/NK progenitors could also be subdivided into two subsets as indicated by the reanalysis of our previously published dataset (Fig. 1A) (27). One progenitor subset expressed genes characteristic of the ILC1/NK cell lineage, including *Klr1b1c* (encoding NK1.1, CD161) *Klrc1* (encoding NKG2A), *Klrc2* (encoding NKG2C), *Klrd1* (encoding CD94), *Ccl5* (encoding CCL5, C-C Motif Chemokine Ligand 5), *Xcl1* (encoding XCL1, X-C Motif Chemokine Ligand 1), but not *Rorc* (encoding RORγt, RAR-related orphan receptor gamma t) (12, 51–53). By contrast the other subset lacked transcripts for these NK cell-related genes but expressed *Rorc* and *Rora* (encoding RORα), transcription factors expressed by transILCPs that are likely committed to the ILC3 lineage (27, 54) (Fig. 1A). Notably, the NKG2A and NKG2C C-type lectin receptor family members associate with CD94 to form heterodimers that mediate responses to infection and tumors (55–57). Further examination of this scRNAseq dataset revealed that the subset composed of cells expressing transcripts for *Klrc1*, *Klrc2*, and *Klrd1*, also coexpressed *Cd244*, *Cd27*, *Il2rb*, *Sell* (encoding CD62L), *Ifngr1* or *Cd226* (encoding DNAM-1) all genes related to the ILC1/NK cell lineage (Fig. 1A) (12, 21, 23, 50, 51, 58, 59).

The expression of NK cell markers on cells within this mixed population of hematopoietic progenitors suggested the presence of committed precursors of NK cells such as pre-NKPs or rNKPs (3, 21, 23). To investigate and verify this possibility, we assessed the expression of the proteins NKG2A, NKG2C, and CD94 by transILCPs coexpressing combinations of ILC transcription factor reporters for *Id2*^+/BFP^, *Gata3*^+/hCD2^, *Rora*^+/Teal^, *Bcl11b*^+/tdTomato^, or *Rorc*^+/Katushka^ in 5× polychromILC mice (15, 27). Our strategy focussed on a bone marrow hematopoietic progenitor population expressing IL-7Rα and Id2, but lacking expression of lineage markers (CD3, CD4, CD5, CD8, CD11b, CD11c, CD19, FcεRI, Ly6C/6G, NK1.1, Ter119) or CD25 (Lin^−^Id2^+^IL-7Rα^+^CD25^−^) (SI Appendix, Fig. S1A), which we had shown previously to contain ILCPs with conserved ILC1/NK cell potential regardless of α4β7, Gata3, or RORγt expression SI Appendix, Fig. S1C (27). Flow cytometry analysis using antibodies against CD94, NKG2A or NKG2A, NKG2C, and NKG2E in combination (an antibody simultaneously recognizing all three receptors, NKG2A/C/E) revealed a fraction of NKG2A/C/E^+^ cells within the Lin^−^Id2^+^IL-7Rα^+^CD25^−^α4β7^−^ population (Fig. 1B). The combined staining of NKG2A/C/E provided the best resolution and was used for subsequent studies.

The Lin^−^Id2^+^IL-7Rα^+^CD25^−^α4β7^−^NKG2A/C/E^+^ transILCPs also lacked expression of *Rorc*-Kat, Gata3-hCD2, and Rora-Teal (Fig. 1C), thereby mirroring the single-cell gene-expression profile, further excluding their relatedness to ILC2 and ILC3 (Fig. 1A). Interestingly, the polychromic reporter mice allowed further resolution, allowing this cell population to be subdivided into Bcl11b-positive and Bcl11b-negative cells, similar to the distribution of NKG2A/C/E^+^ ILC1 (Bcl11b-positive) and NK cells (Bcl11b-negative) in liver lymphocytes (Fig. 1C and SI Appendix, Fig. S1B). Based on this phenotype, NKG2A/C/E^+^ progenitors mapped onto ILCP transitional states defined as IVb and III^lo^ in our previous study, which indeed contained transILCPs with ILC1/NK developmental potential (SI Appendix, Fig. S1C) (27). Interestingly, we noticed that while a proportion NKG2A/C/E^+^ transILCPs expressed Bcl11b, their expression was lower than the expression displayed by NKG2A/C/E^−^ transILCPs (Fig. 1C). Further analysis indicated that NKG2A/C/E^+^ transILCPs, regardless of their Bcl11b expression, expressed NKG2D (encoded by *Klrk1*), CD122 (*Il2rb*), and CD244 (also known as 2B4, SLAMF4), but lacked surface CD49b (DX5, *Itga2*) (SI Appendix, Fig. S1 D and E), suggesting a phenotype similar to rNKPs (Lin^−^IL-7Rα^+^CD244^+^CD122^+^) (3, 23, 24). Our previous gene expression analysis had also suggested that the mix of putative ILC1, ILC3, and NKPs expressed *Tbx21* transcripts (Fig. 1A). To determine T-bet expression, we used a newly generated reporter mouse strain in which the human CD4 (hCD4) protein is produced downstream of a P2A sequence at the C terminus of the T-bet protein (T-bet^+/hCD4^ mice; SI Appendix, Fig. S2 A and B). We confirmed that surface hCD4 staining coordinated with T-bet expression detected using ICS in splenic NK cells (SI Appendix, Fig. S2C) and other lymphocytic populations in the lymph nodes, lung, and small intestine lamina propria (siLP) (SI Appendix, Fig. S2 D–F). In addition, T-bet-hCD4 reporter mice did not present apparent phenotypic abnormalities (SI Appendix, Fig. S2 G–I). We intercrossed the T-bet-hCD4 reporter mice with our 5× polychromILC mouse to generate an *Id2*^+/BFP^, *Gata3*^+/hCD2^, *Rora*^+/Teal^, *Bcl11b*^+/tdTomato^, *Rorc*^+/Katushka^, and *Tbx21*^+/hCD4^ 6× polychro-mILC mice. Lin^−^Id2^+^IL-7Rα^+^CD25^−^NKG2A/C/E^+^ transILCPs were found to be uniformly positive for T-bet-hCD4 (Fig. 1D), further suggesting their developmental relationship to ILC1 and NK cells (11, 22, 43).

Notably, NKG2A/C/E^+^ transILCPs did not express surface CD49b (SI Appendix, Fig. S1E) or NK1.1 proteins (despite expressing *Klr1b1c* mRNA; N.B. NK1.1^+^ cells were excluded with the Lin^+^ cells) indicating that they had yet to differentiate into NK cells (21, 22, 32). However, the presence of NKG2D raised the possibility that these bone marrow-resident NKG2A/C/E^+^ cells constituted an “immature” or “underdeveloped” NK cell as opposed to an ILC progenitor (14, 22, 35, 52, 60, 61). To test this possibility, we analyzed the expression of two additional NK maturation markers, Eomes and perforin, ex vivo by intracellular cytokine analysis (ICS). While splenic and hepatic Lin^−^NK1.1^+^ cells displayed high levels of perforin and Eomes, and even a fraction of BM NK1.1^+^ cells were positive for both molecules (likely BM iNKs), Lin^−^Id2^+^IL-7Rα^+^CD25^−^NKG2A/C/E^+^ cells were negative for Eomes and perforin and more closely resembled an ILCP (SI Appendix, Fig. S1F). We also assessed PLZF (encoded by *Zbtb16*) expression which is known to reduce as ILCPs develop and commit toward more lineage-restricted states during their maturation process (26–28, 31). To assess PLZF expression, we used a newly developed Citrine reporter mouse strain (SI Appendix, Fig. S3 A and B) similar to the *Zbtb16*-tdTomato reporter line published recently (27). *Zbtb16*-Citrine (*Zbtb16*^+/Citrine^) mice did not display any noticeable phenotypic variation, and Citrine was a faithful reporter of PLZF detected by ICS (SI Appendix, Fig. S3C–H). We interrogated the expression of PLZF in *Id2*^+/BFP^, *Zbtb16*^+/Citrine^, *Bcl11b*^+/tdTomato^, and *Rorc*^+/Katushka^ 4× polychromILC mice. While the Lin^−^Id2^+^IL-7Rα^+^CD25^−^NKG2A/C/E^−^ ILCP population contained cells displaying a range of PLZF expression (which would include the cells previously known as CHILP with the highest PLZF expression), Lin^−^Id2^+^IL-7Rα^+^CD25^−^NKG2A/C/ E^+^ transILCPs, irrespective of Bcl11b expression, were PLZF^lo/int^ (Fig. 1E).

Taken together these data suggest that the Lin^−^Id2^+^IL-7Rα^+^CD25^−^α4β7^−^NKG2A/C/E^+^T-bet^+^Eomes^−^PLZF^lo/int^ transILCPs are more restricted to the ILC1/NK cell lineage. Notably, however, the phenotype of these cells did not fit with the canonical NK cell ontogeny (3, 24). For example, they expressed IL-7Rα, CD122, NKG2D, and CD244 similar to pre-NKPs/rNKPs (21, 23), but also expressed NKG2A/C/E like iNKs, which lack IL-7Rα (60).

### NKG2A/C/E^+^Bcl11b^−^ TransILCPs are BM Progenitors Exclusively Committed toward the Type 1 ILC/NK Cell Lineage

**Fig. 2:**
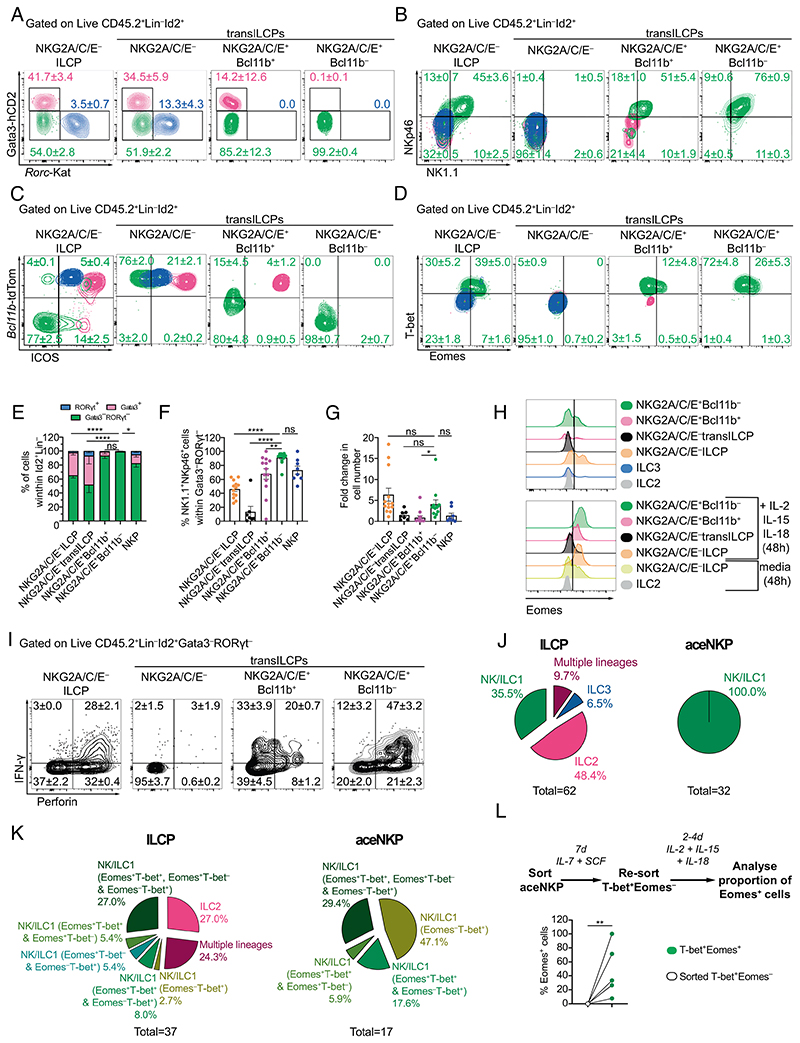
NKG2A/C/E expression identifies hematopoietic progenitors exclusively committed to ILC1/NK lineage. NKG2A/C/E^−^ ILCPs, NKG2A/C/E^−^ transILCPs, NKG2A/C/E^+^Bcl11b^−^ transILCPs, and NKG2A/C/E^+^Bcl11b^+^ transILCPs, as defined in SI Appendix, Fig. S4 B and C, were FACS-sorted from bone marrow (femora, tibiae, and ilia) of 5× polychromILC reporter mice an in vitro cultured on mitomycin C-treated OP9 stromal cells in the presence of IL-7 and SCF for 7 d. (*A*) Contour plots of Gata3^+^(pink), RORγt^+^ (blue) and Gata3^−^RORγt^−^ (green) cells after 7 d of culture (gated on Live CD45^+^Id2^+^Lin^−^ cells as indicated in SI Appendix, Fig. S4D). Numbers indicate frequencies of their respective colored population as mean ± SEM. (*B*–*D*) Expression of NK1.1, NKp46, Bcl11b, ICOS, T-bet, and Eomes in cells from (*A*). Numbers indicate frequencies in each gate within Gata3^−^RORγt^−^ lymphocytes as mean ± SEM. (*E*–*G*) Cumulative data indicating proportions of Gata3^+^, RORγt^+^, and Gata3^−^RORγt^−^ cells (*E*), NKp46^+^NK1.1^+^ cells (*F*), and cell number increase in the indicated populations (*G*). (*H*) Representative histograms depicting Eomes expression on cells derived from the indicated progenitors, ILC2s or ILC3s at day 7 (*Upper*), or after a further 48-h stimulation with IL-2, IL-15, and IL-18 (*Bottom*) of in vitro culture. (*I*) Cells from (*A*) were further cultured for 48 h in the presence of IL-2, IL-15, and IL-18, and protein transport inhibitor for the last 4–6 h. Expression of perforin and IFN-γ in Gata3^−^RORγt^−^ cells. (*J* and *K*) Characterization of progeny derived from clonal analysis of single NKG2A/C/E^−^ ILCPs and aceNKPs, purified from the bone marrow of 5× polychromILC mice, after coculture with OP9 stromal cells for 22 d (*J*) or 14 d (*K*) (*J*: NK/ILC1 = Id2^+^Gata3-hCD2^−^*Rorc*-Kat^−^NK1.1^+^NKp46^+^*Bcl11b*-tdTom^−^Rora-Teal^−^; ILC2 = Id2^+^Gata3-hCD2^+^*Rorc*-Kat^−^*Bcl11b*-tdTom^+^Rora-Teal^+^; ILC3 = Id2^+^Gata3-hCD2^−^*Rorc*-Kat^+^; *K*: NK/ILC1 = Id2^+^Gata3-hCD2^−^*Rorc*-Kat^−^NK1.1^+^Eomes^+^ and/or T-bet^+^; ILC2 = Id2^+^Gata3-hCD2^+^*Rorc*-Kat^−^*Bcl11b*-tdTom^+^NK1.1^−^; ILC3 = Id2^+^Gata3-hCD2^−^*Rorc*-Kat^+^). Numbers below the pie charts indicate the total number of viable progenitors that differentiate into ILC-lineages: J, ILCP 62/126, aceNKP 32/98; K, ILCP: 37/48, aceNKP: 17/28. (*L*) *Eomes*-GFP^+^ cells were analyzed after re-sorted T-bet-hCD4^+^*Eomes*-GFP^−^ aceNKP-derived progeny were cultured with IL-7, IL-2, IL-15, IL-18, and SCF on OP9 stromal cells for 4 d. aceNKPs were isolated from *Id2*^+/BFP^, *Bcl11b*^+/tdTomato^, *Rorc*^+/Katushka^, *Eomes*^+/GFP^ and *Tbx21*^+/hCD4^ mice. Representative and cumulative data from one (*D*, n = 3; *K*, n = 28-48), two (*C*, n = 5; *J*, n = 66–122; *L*, n = 6), or 4–5 experiments (*A*, *B*, *E*–*I*, n = 8–13). Numbers depicted in panels indicate frequency of cells. Data presented as mean ± SEM. ns: no significant; *P < 0.05; **P < 0.01; ****P < 0.0001; two-way (*E*), one-way (*F*), or Brown-Forsythe and Welch (*G*) ANOVA with Dunnett’s multiple comparisons test.

To determine the lineage potential of Lin^−^Id2^+^IL-7Rα^+^CD25^−^α4β7^−^NKG2A/C/ E^+^T-bet^+^Eomes^−^PLZF^lo/int^ transILCPs, we assessed their lineage potential in vitro. Based on the phenotypic features of these progenitor populations, we hypothesized that the NKG2A/C/E^−^progenitors would contain ILCPs and transILCPs (characterized by variable levels of PLZF expression) restricted to helper ILC lineages based on their Gata3 expression profile (Gata3^hi^ transILCPs being committed to ILC2 lineage), while the NKG2A/C/E^+^ progenitors would be biased to an ILC1/NK cell lineage (SI Appendix, Fig. S4A). Lin^−^Id2^+^IL-7Rα^+^CD25^−^ cells that were either NKG2A/C/E^−^ ILCPs, NKG2A/C/E^+^Bcl11b^+^ transILCPs, or NKG2A/C/E^+^Bcl11b^−^ transILCPs were sorted in parallel from bone marrow and cultured on OP9 stromal cells with IL-7 and stem cell factor (SCF) for 7 d before determining the phenotypes of their respective progeny (Fig. 2 and SI Appendix, Fig. S4 B and C). We observed that the NKG2A/C/E^+^Bcl11b^+^ transILCP subset was equivalent to the NKG2A/C/E^+^ fraction of our previously defined population III^lo^ (Lin^−^Id2^+^IL-7Rα^+^CD25^−^Bcl11b^+^Gata3^−^RORγt^−^ transILCPs, SI Appendix, Fig. S4 B and C) (27). Thus, in addition to the three aforementioned progenitor populations, we sorted and cultured the NKG2A/C/E^−^ III^lo^ transILCPs (hereafter called NKG2A/C/E^−^ transILCPs) to test whether removing the NKG2A/C/E^+^ progenitors from this population would preclude the appearance of ILC1/NK cells.

We analyzed the progeny of these four sorted progenitor populations after 7 d in culture and defined the resulting ILC lineages by the differential expression of Gata3, RORγt, NK1.1, NKp46, Bcl11b, ICOS, T-bet, and Eomes. ILC1/NK cells were characterized as Gata3^−^RORγt^−^NK1.1^+^NKp46^+^ICOS^−^Bcl11b^−^T-bet^+^Eomes^–/+^; ILC2s as Gata3^+^RORγt^−^NK1.1^−^NKp46^−^ICOS^hi^Bcl11b^+^T-bet^−^Eomes^−^; and ILC3 as Gata3^−^RORγt^+^NK1.1^−^NKp46^–/+^ICOS^int^ Bcl11b^+^T-bet^−^Eomes^−^. We determined that NKG2A/C/E^−^ ILCPs gave rise to all the ILC1/NK cell, ILC2, and ILC3 populations, while the NKG2A/C/E^+^Bcl11b^+^ transILCPs were more restricted producing ILC1/NK cells and ILC2s, whereas the NKG2A/C/E^+^Bcl11b^−^ progenitors were singularly committed to the ILC1/NK lineage (Fig. 2 A–F and SI Appendix, Fig. S4D).NKG2A/C/E^−^ transILCPs were incapable of producing ILC1/NK cells, but continued to give rise to ILC2s and ILC3s, confirming that the NKG2A/C/E^+^ cells represented the ILC1/NK progenitors identified among III^lo^ transILCPs (27). Notably, comparison to previously defined pre-NKPs/rNKPs (Id2^+^NKP: Lin^−^IL-7Rα^+^ CD122^+^Id2^+^ cells, that we defined here with the help of the Id2-BFP reporter; henceforth referred to as NKPs; SI Appendix, Fig. S4E) (21, 23) indicated that these NKPs still retained ILC2 and ILC3 potential (Fig. 2E) and that only ~70% of their progeny were NK cells, as compared to ~100% of the progeny from the NKG2A/C/E^+^Bcl11b^−^ progenitors (Fig. 2F). We noticed that NKPs identified with the Id2-BFP could be further subdivided into Id2-BFP^hi^ and Id2-BFP^int^ populations containing CD25-positive and Bcl11b-positive progenitors, likely explaining their residual capacity to differentiate into ILC2s (SI Appendix, Fig. S4E). Similar to pre-NKPs/rNKPs, NKG2A/C/E^−^ ILCPs, NKG2A/C/E^+^Bcl11b^+^ tran-sILCPs and NKG2A/C/E^+^ Bcl11b^−^ transILCPs lacked expression of Flt3 (CD135) and had variable degrees of c-Kit and Sca1 expression (SI Appendix, Fig. S4F) (21). The NKG2A/C/E^+^Bcl11b^−^ tran-sILCPs were also considerably more proliferative than their NKG2A/C/E^+^Bcl11b^+^ counterparts, which proliferated poorly in vitro (Fig. 2G). ILCPs, NKPs, or NKG2A/C/E^–/+^ transILCPs cultured with IL-7 and Flt3L (Fms Related Receptor Tyrosine Kinase 3 Ligand) on OP9 or OP9-DL4 stromal cells did not produce B or T cells, in contrast to CLPs, which retained T and B cell potential (SI Appendix, Fig. S4 G and H).

Using an alternative phenotyping approach to define pre-NKPs/rNKPs (23), we sought to refine their relationship with NKG2A/C/E^+^Bcl11b^−^ transILCPs (hereafter called aceNKPs due to their expression of NKG2A/C/E). The frequency and number of aceNKPs in the bone marrow was lower than pre-NKPs and rNKPs (SI Appendix, Fig. S4I), and we determined that a similar NKG2A/C/E^+^ subset existed as a minority within the rNKP population. However, rNKPs also contained cells resembling ILCP, with a mix of Bcl11b^–/+^, CD25^–/+^ and Id2^int/+^ cells (SI Appendix, Fig. S4 J–L). Although most aceNKPs expressed CD27 and CD244 (SI Appendix, Fig. S4 K and L), similar to rNKP, some had lower levels of CD27, suggesting that aceNKPs may not completely overlap phenotypically with the rNKPs. Notably, pre-NKPs/rNKPs cultured on OP9 cells in the presence of IL-15 as indicated in the original reports (21, 23), completely matured into NK cells as defined by NK1.1 and Eomes expression, but differentiated into ILC1/NK cells and ILC2s in its absence, suggesting that the ILC2/ILC3 potential is eclipsed by IL-15 (SI Appendix, Fig. S4 M and N).

Next, we investigated the capacity of the ILC1/NK cell populations derived from the different progenitor subsets to express surface markers or secreted products characteristic of NK cells or ILC1, following stimulation. After 7 d of culture with IL-7 and SCF, we added IL-2, IL-15, and IL-18 for 48 h to activate/mature the cells. Strikingly, the aceNKPs became uniformly Eomes^+^CD49a^−^CD49b^+^CD122^+^CD90^lo/hi^KLRG1^int^Ly49C/F/I/H^+^ and produced predominantly perforin and IFN-γ, likely highlighting their greater predisposition to generate fully mature cytotoxic NK cells rather than ILC1s (Fig. 2 H and I and SI Appendix, Fig. S4O). By contrast NKG2A/C/E^+^Bcl11b^+^ transILCPs gave rise to Eomes^lo^CD49a^+/–^CD49b^+/–^CD122^+^CD90^hi^KLRG1^int^Ly49C/F/H/I^lo^ cells and produced IFN-γ and less perforin (Fig. 2 H and I and SI Appendix, Fig. S4O). These data suggest that the expression of Bcl11b correlates with the commitment of NKG2A/C/E^+^ transILCPs to an ILC1-like cytokine-biased state where the differentiating progenitors acquire a helper ILC phenotype (IFN-γ-producing ILC), while NKG2A/C/E^+^ transILCPs lacking Bcl11b mature into IFN-γ and perforin-positive cytotoxic innate lymphocytes.

We performed single-cell differentiation analysis to determine if further heterogeneity existed within aceNKPs. Compared with NKG2A/C/E^−^ ILCPs that contained progenitors committed to ILC1/NK cells, ILC2s, ILC3s, or multiple lineages (Fig. 2 J and K and SI Appendix, Fig. S4 P and Q), clonal analysis of aceN-KP-derived progeny showed that they homogenously became ILC1/NK cells (Fig. 2J and SI Appendix, Fig. S4P) expressing T-bet or a mix of cells expressing T-bet and/or Eomes (Fig. 2K and SI Appendix, Fig. S4Q). The differentiation of aceNKPs into T-bet^+^Eomes^−^ and/or T-bet^+^Eomes^+^ cells (Fig. 2 D and K) together with the observation that the provision of IL-2, IL-15, and IL-18 made the aceNKP progeny homogenously express Eomes (Fig. 2H) suggested that T-bet^+^Eomes^−^ cells may subsequently up-regulate Eomes upon maturation. To test this hypothesis, we additionally cultured aceNKPs from *Id2*^+/BFP^, *Bcl11b*^+/tdTomato^, *Rorc*^+/Katushka^, *Eomes*^+/GFP^ and *Tbx21*^+/hCD4^ mice for 7d prior to resorting the T-bet^+^Eomes^−^ progeny, which we then recultured with IL-2, IL-15, and IL-18 for a further 2–4 d before analysis. We found that T-bet^+^Eomes^−^ cells can up-regulate Eomes upon provision of maturation signals (Fig. 2L). We observed a significant upregulation of *Eomes*-GFP (Fig. 2L) indicating that aceN-KP-derived Tbet-hCD4^+^*Eomes*-GFP^−^ ILC1/NK cells may require further time or extra maturation signals to express Eomes.

Together these results demonstrate that aceNKPs (Lin^−^Id2^+^IL-7Rα^+^CD25^−^NKG2A/C/E^+^Bcl11b^−^ cells) are highly lineage restricted ILC1/NKPs that cannot produce other ILC subsets. Purification of aceNKPs removed the residual ILC2 and ILC3 lineage capacity that remained in previously reported isolation strategies (21, 23), and identified potential ILC1Ps within the Bcl11b^+^ counterpart of the NKG2A/C/E^+^ transILCPs.

### aceNKPs Produce ILC1/NK Cell Progeny Regardless of the Tissue Microenvironment

**Fig. 3:**
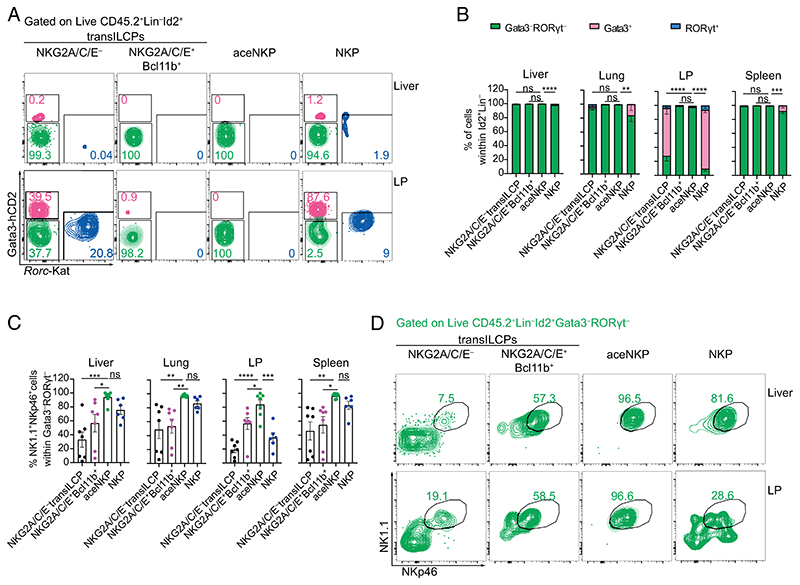
aceNKP (NKG2A/C/E^+^Bcl11b^−^ transILCP) progeny is restricted to ILC1/NK cells regardless of tissue microenvironment. Approximately 300–600 of the specified precursor cells were intravenously adoptively transferred into sublethally irradiated *Rag2*^–/–^*Il2rg*^–/–^ mice. Indicated tissues were analyzed for the presence of ILC progeny after 6–7 wk. (*A*) Representative contour plots from the liver and siLP depicting proportions of Gata3^+^ (pink), RORγt^+^ (blue), and Gata3^−^RORγt^−^ (green) lymphocytes derived from adoptively transferred NKG2A/C/E^−^ transILCPs, NKG2A/C/E^+^Bcl11b^+^ transILCPs, aceNKPs or NKPs. (*B*) Frequencies of Gata3^+^, RORγt^+^, and Gata3^−^RORγt^−^ donor cells within the mononuclear cell fraction from the liver, lung, lamina propria, and spleen (gated of Live CD45.2^+^ID2^+^Lin^−^ cells). (*C*) Frequencies of NK1.1^+^NKp46^+^ cells within Gata3^−^RORγt^−^ lymphocytes from (*A* and *B*). (*D*) Representative contour plots from the liver and siLP depicting proportions of ILC1/NK cells (NK1.1^+^NKp46^+^) within Gata3^−^RORγt^−^ (green) lymphocytes from (*A*). Representative plots and pooled cumulative data from two independent experiments (*A*–*D*, n = 6–7 animal/group). Numbers depicted in panels indicate frequency of cells. Cumulative data represented as mean ± SEM. ns: no significant; *P< 0.05; **P< 0.01; ***P< 0.001; ****P< 0.0001; two-way (*B*) or one-way (*D*) ANOVA with Dunnett’s multiple comparisons test.

Variable NK cell phenotypes have been reported to be dependent on the tissue microenvironments in which they develop or mature (52, 53, 62–65). To determine the in vivo cellular development of purified NKG2A/C/E^−^ and NKG2A/C/E^+^Bcl11b^+^ transILCPs, aceNKPs and NKPs, these cell populations (CD45.2) were adoptively transferred intravenously into sublethally irradiated *Rag2*^–/–^*Il2rg*^–/–^ (CD45.1) mice. With the exception of NKG2A/C/E^−^ transILCPs, all these progenitor subsets gave rise to ~100% ILC1/NK cells in the liver and spleen (Fig. 3 A–D and SI Appendix, Fig. S5 A and B) ~6 wk after adoptive transfer. However, the NKP cells (mix of pre-NKPs and rNKPs) gave rise to ~20% ILC2 in the lungs and almost 80% ILC2 and 5% ILC3 in the siLP (Fig. 3 A and B). Surprisingly, adoptive transfer of purified rNKPs also gave rise to ILC2s and ILC3s in the intestinal lamina propria (SI Appendix, Fig. S5C). By contrast aceNKPs produced only ILC1/NK cells in the lung and lamina propria (Fig. 3 A and C). Furthermore, analysis of NK1.1 and NKp46 expression within the Lin^−^CD45.2^+^Id2^+^Gata3^−^RORγt^−^ gate in the lamina propria indicated that ILC1/NK cells only represented approximately 50% of these cells arising from the NKPs (Fig. 3 C and D), as compared with nearly 100% produced by the aceNKPs (Fig. 3 C and D). Interestingly, Lin^−^CD45.2^+^Id2^+^Gata3^−^RORγt^−^ cells that lacked NK1.1 and NKp46, and derived from NKG2A/C/E^−^ transILCPs and NKPs, expressed more RORα, Bcl11b, and IL-7Rα than their NK1.1^+^NKp46^+^ counterparts, suggesting similarities with ILC1s, ILC2s, and ILC3s (SI Appendix, Fig. S5D) (27).

Ex vivo stimulation of cells from liver samples with IL-2, IL-15, and IL-18, indicated that aceNKPs became consistently mature ILC1/NK cell-like perforin and IFN-γ coproducers (SI Appendix, Fig. S5E), while NKG2A/C/E^+^Bcl11b^+^ progenitors gave rise to predominantly IFN-γ-producing cells that were negative for perforin (SI Appendix, Fig. S5E), in line with an ILC1-like phenotype. While approximately 50% of the NKP progeny coexpressed IFN-γ and perforin, almost 40% were negative for these factors (SI Appendix, Fig. S5E). IL-2, IL-15, and IL-18 ex vivo stimulation promoted the upregulation of T-bet and Eomes in the liver cells derived from all the transferred progenitor populations. However, aceNKP and NKP progeny displayed higher levels of Eomes consistent with progenitors giving rise to mature NK cells (SI Appendix, Fig. S5F).

Collectively, these data confirm that aceNKPs (i.e. NKG2A/C/E^+^Bcl11b^−^ transILCPs) give rise exclusively to mature ILC1/NK cells. Importantly, the identification of aceNKPs allows for the discrimination of committed ILC1/NK precursors away from more heterogeneous ILC progenitors (21, 23, 50).

### aceNKPs Represent a Common Progenitor of Cytotoxic and Helper ILC1/NK Cells

To understand whether aceNKP cells were committed to NK cells or a mix of group 1 ILCs including helper and cytotoxic ILC1s and NK cells, we performed multiparametric flow cytometry and scRNAseq on the in vivo progeny of aceNKP cells.

**Fig. 4:**
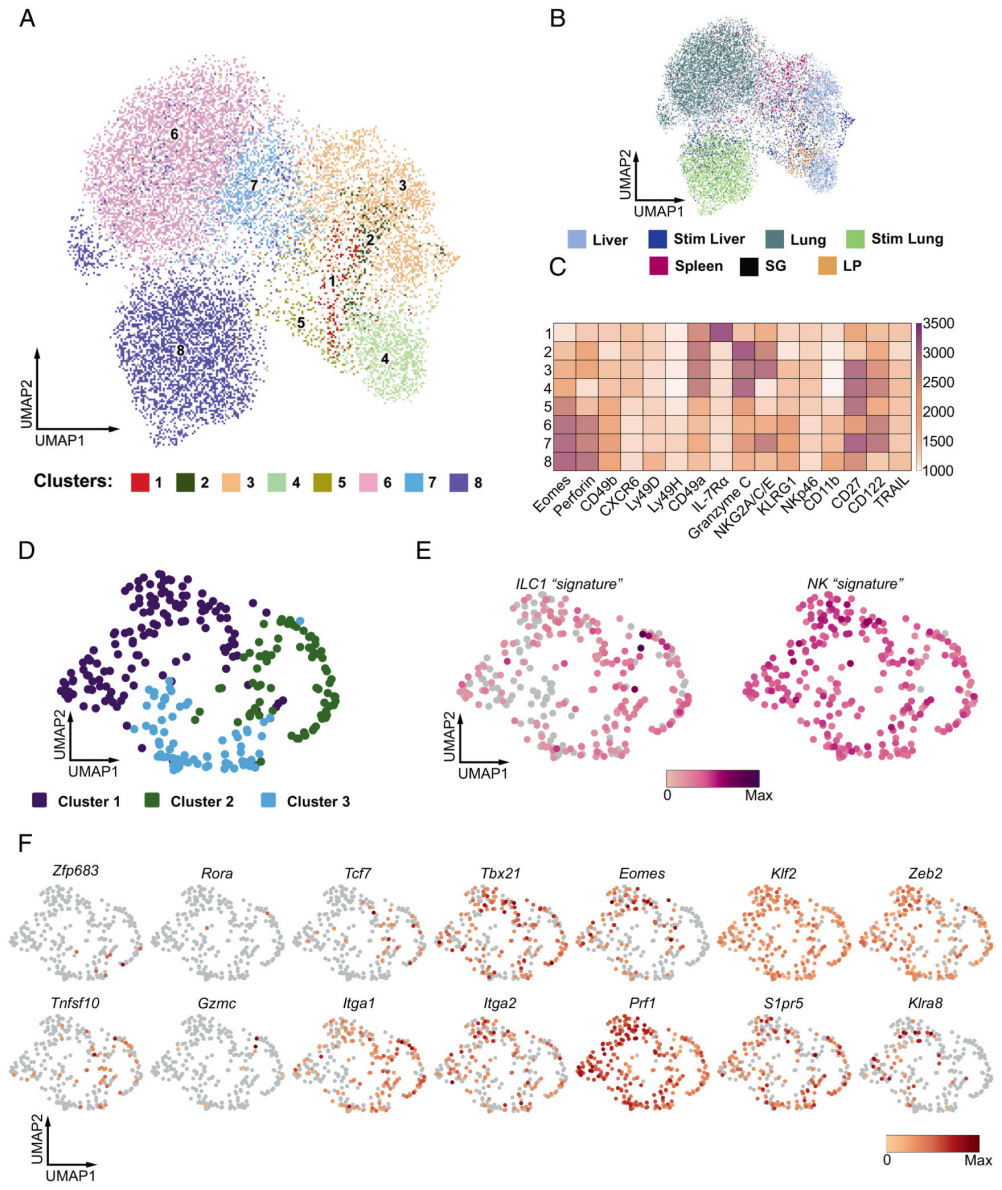
aceNKPs represent a common progenitor of cytotoxic and helper ILC1/NK cells. Approximately 100 aceNKPs were intravenously adoptively transferred into sublethally irradiated *Rag2*^–/–^*Il2rg*^–/–^ mice. Indicated tissues were analyzed for the expression of ILC1/NK cell markers within aceNKP-derived progeny after 6–7 wk. (*A*–*C*) Results from high-dimensional spectral flow cytometry analysis of aceNKP-derived progeny from several tissues. (*A*) Uniform manifold approximation and projection (UMAP) visualization of in vivo aceNKP-derived progeny colored by cluster. (*B*) UMAP visualization of in vivo aceNKP-derived progeny colored by tissue of origin. (*C*) Heatmap for expression of ILC1/NK cell markers within the identified clusters. Color represents maximum-normalized mean intensity expression of the indicated proteins within each cluster. (*D*–*F*) Results from scRNAseq analysis of aceNKP-derived progeny in the lung. (*D*) UMAP visualization of the lung aceNKP-derived progeny colored by graph-based clusters. (*E*) UMAP plots depicting manually curated ILC1 (*Left*) and NK cell (*Right*) transcriptional signatures from cells in *D*. (*F*) UMAP plots of representative gene expression. Genes shown serve to discriminate between ILC1s and NK cells. Data shown are pooled from one (*D*–*F* = 3) or two/three independent experiments (*A*–*C* = 4–7 animal/tissue).

We implemented high-dimensional spectral flow cytometry to comprehensively probe the progeny of aceNKP for the expression of proteins traditionally used to differentiate between ILC1s and NK cells (Fig. 4 A–C and SI Appendix, Fig. S6 A–D; NK markers: Eomes, perforin, CD49b, Ly49D, Ly49H, KLRG1, CD11b; ILC1 markers: CD49a, IL-7Rα, granzyme C, TRAIL, CXCR6, Common markers: CD122, CD27, NKG2A/C/E, NKp46). Unsupervised analysis of flow cytometry data for aceNKP-derived donor cells isolated from the liver, lung, spleen, salivary glands (SG) and lamina propria identified eight clusters (Fig. 4A) spanning these five tissues to variable degrees (Fig. 4B and SI Appendix, Fig. S6 A and B). We included liver and lung lymphocytes that were pulse-stimulated with IL-2, IL-15 and IL-18 for 5 h.

Strikingly, instead of clearly defined clusters of ILC1 and NK cells expressing their associated markers (Fig. 4C and SI Appendix, Fig. S6 C and D), we observed that unsupervised dimensionality reduction and clustering suggested a continuum of Eomes expression and differential coexpression of CD49a and CD49b in certain clusters. At one end of this spectrum, we could identify cluster (C) 1: Eomes-negative lymphocytes with coexpression of CD49a, IL-7Rα and CXCR6, which were negative for granzyme C, identified recently as a characteristic marker of ILC1s (66). At the other end, C8 showed the highest expression of Eomes together with perforin, CD49b, CD11b, KLRG1 and some levels of Ly49D and Ly49H. While C1 resembled helper ILC1s, and C8 mature cytotoxic NK cells, we noted that C5 and C7 (two of the four clusters with the highest Eomes and CD49b expression) also expressed some degree of CD49a and granzyme C (Fig. 4C) failing to match the phenotype of ILC1s or NK cells. Furthermore, while C2–C4 resembled cytotoxic ILC1s because they coexpressed CD49a, CXCR6 and granzyme C but not IL-7Rα (12), C3 and C4 also expressed Eomes, KLRG1 and some level of CD49b. Surprisingly, we did not detect the expression of TRAIL in any of the aceNKP progeny populations, including those more ILC1-like clusters (Fig. 4C and SI Appendix, Fig. S6 C and D). While the cells integrating these clusters were not confined to single tissues, we noticed that SG and intestine were enriched in C1 and C2 cells, suggesting that tissue-specific microenvironmental cues can shift the balance of the observed ILC1/NK spectrum (SI Appendix, Fig. S6 A–C). Interestingly, ex vivo stimulated progeny from the liver and lung were enriched in C8 indicating that they can mature upon suitable cues such as IL-15 (SI Appendix, Fig. S6 A–C). Thus, these results suggested that aceN-KPs can develop into a continuum of cytotoxic and helper ILC1/ NK cells.

scRNA sequencing of the aceNKP progeny in the lung confirmed that aceNKPs developed into a spectrum of group 1 ILCs with cytotoxic and helper phenotypes, confirming that aceNKPs are common progenitors of ILC1/NK cells rather than NK cells alone (Fig. 4 D–F). The similarity between the multiple cells made it difficult to distinguish clear subclusters. Graph-based clustering defined three clusters (Fig. 4D). However, these were fairly similar only being defined by the differential expression of 10 genes (*Serpinb9b*, *Klra5*, *Klra7*, *Klra8*, *Klra9*, *Klri2*, *Itgam*, *Hsph1*, *Cd7*, and *Ltb*; SI Appendix, Fig. S6E).Consequently, we were unable to separate the aceNKP-progeny according to the traditional dichotomy between ILC1s and NK cells. Indeed, using sets of manually curated genes that are reported to differentiate ILC1 (signature genes - *Cd3g*, *Cxcr6*, *Gzmc*, *Ikzf2*, *Il21r*, *Il7r*, *Itga1*, *Itgae*, *Rora*, *Lag3*, *Tnfrsf25*, *Zfp683*) from NK cells (signature genes - *Itga2*, *Klf2*, *Eomes*, *Irf8*, *S1pr5*, *Zeb2*, *Klra8*), regardless of their cytotoxic or helper phenotype (Fig. 4E) (12, 52, 53, 66, 67), we failed to observe clear demarcation of transcriptional profiles that separate the ILC1 and NK cell lineages (Fig. 4 E and F), indicating the difficulty in discriminating between these highly related group 1 innate lymphocytes. As shown in Fig. 4F, only minor groups of cells expressed transcripts classically assigned to ILC1, such as *Tnfsf10* (encoding TRAIL, 14%) or *Zfp683* (encoding the ILC1-related transcription factor Hobit, 2%) (12), while 30 and 49% of the lung cells, respectively, expressed *Eomes* or *S1pr5*, normally used to define NK cells.

These results suggest aceNKPs are common precursors for group 1 ILCs and develop into innate lymphocytes that, rather than clearly segregating into ILC1s and NK cells, represent a continuous spectrum of helper and cytotoxic ILC1 and NK cells.

### aceNKP-Derived ILC1/NK Cells Have Antitumor Activity In Vivo

**Fig. 5:**
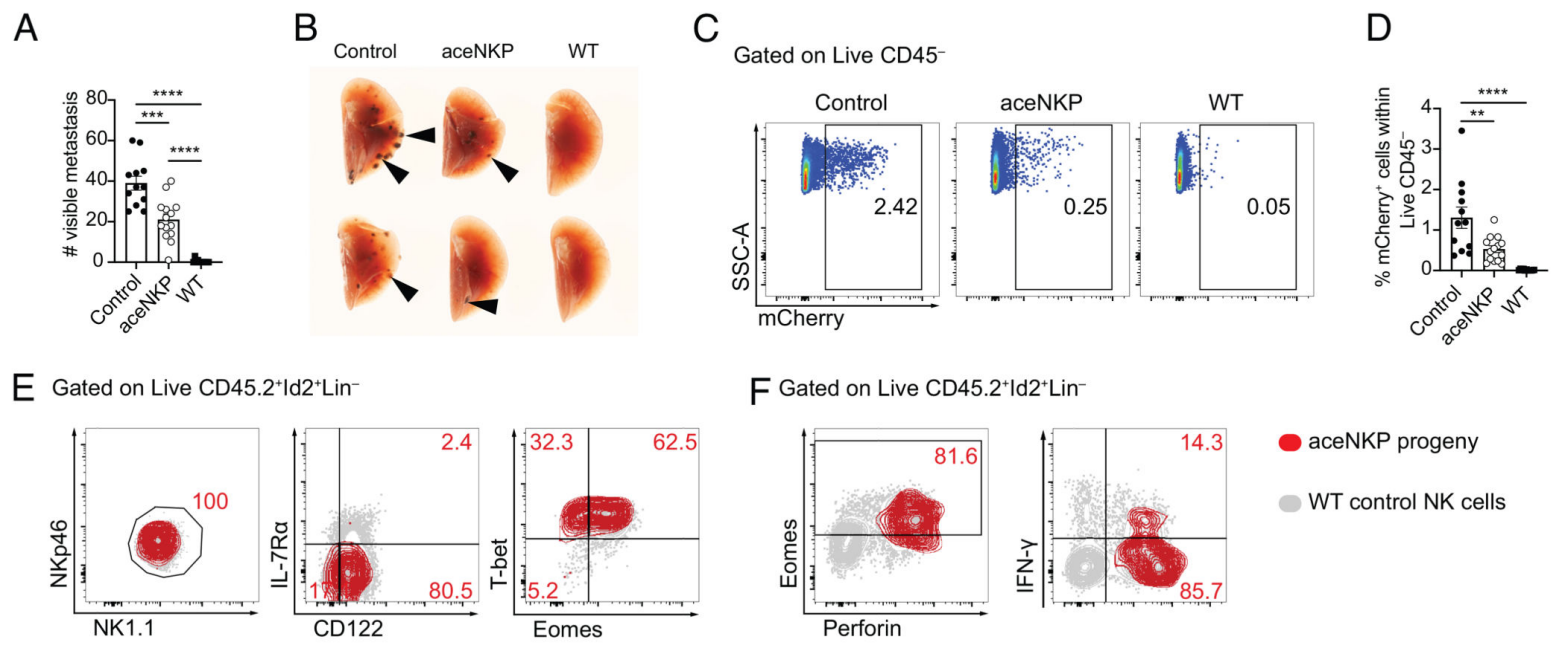
aceNKP progeny present antitumor activity in vivo. Approximately 300–400 aceNKPs were intravenously adoptively transferred into sublethally irradiated *Rag2*^–/–^*Il2rg*^–/–^ mice. 6 wk later, mice were challenged with 10^5^ B16F10-mCherry melanoma cells i.v. and lung metastatic foci were analyzed after 14 d, as indicated in SI Appendix, Fig. S5C. (*A*) Number of metastases in the lung left lobe of *Rag2*^–/–^*Il2rg*^–/–^ mice receiving PBS (Control), or aceNKPs. Sublethally irradiated WT animals were used as immunocompetent control animals. (*B*) Image of representative lungs with metastatic foci from (*A*). Black arrows indicate metastatic foci. (*C* and *D*) Representative dot plots (*C*) and cumulative data (*D*) of the proportion of B16F10 melanoma cells (mCherry^+^) within the right lung lobes (superior, middle, inferior and postcaval lobes). (*E*) Representative contour plots of the phenotype analysis of aceNKP progeny cells in the lungs of tumor-bearing mice, identified as depicted in SI Appendix, Fig. S7B. (*F*) Density-fractioned lung single-cell suspensions were stimulated overnight in the presence of protein transport inhibitors and stained for perforin, IFN-γ, and Eomes. Analysis of aceNKP progeny cells in the lungs of tumor-bearing mice, identified as depicted in SI Appendix, Fig. S7 B and C. Representative and cumulative data from three independent experiments (n = 10–14). Numbers depicted in panels indicate frequency of cells. Data presented as mean ± SEM. **P < 0.01; ***P < 0.001; ****P < 0.0001; one-way ANOVA with Tukey’s multiple comparisons test.

Mature NK cells and cytotoxic ILC1s provide protection against infection and cancer (1, 65, 66). To assess the functional capability of ILC1/NK cells produced from aceNKPs, we examined their ability to protect from cancer metastasis. The C57BL/6 mouse-derived melanoma cell line B16F10 (expressing mCherry) was selected for its capacity to produce metastases in the lung following intravenous injection (68). aceNKPs (CD45.2) were adoptively transferred intravenously into sublethally irradiated *Rag2*^–/–^*Il2rg*^–/–^ (CD45.1) recipients and after ~6 wk these mice were challenged with the B16F10 melanoma cell line (SI Appendix, Fig. S7A). After 14 d cancer metastases in the lung were counted. Reconstitution of recipients with aceNKPs resulted in a marked reduction in visible lung tumors as compared with control animals that did not receive cells (Fig. 5 A and B). Immunocompetent sublethally irradiated wild-type (WT) animals completely controlled the formation of metastases. The antitumor activity promoted by aceNKP-derived ILC1/NK cells was confirmed by the decreased proportion of mCherry^+^ tumor cells in the lungs of reconstituted mice as compared with controls (Fig. 5 C and D). Following a short stimulation with PMA/ionomycin in vitro, analysis of Lin^−^CD45.2^+^Id2^+^ lymphocytes from the lungs of the aceNKP recipient mice showed that the majority acquired a NK-like phenotype (NK1.1^+^NKp46^+^CD122^+^IL-7Rα^−^T-bet^+^Eomes^+^) and coexpressed perforin (though only ~15% were IFN-γ-positive) in the lung tumor microenvironment (Fig. 5 E and F and SI Appendix, Fig. S7 B and C). Thus, aceNKP-derived NK cells (and possibly ILC1s) act functionally to combat tumor metastasis.

## Discussion

The recent development of multicolor ILC transcription factor reporter mice, combined with flow cytometric analysis of cell surface receptor expression and single-cell RNA sequencing, has facilitated far greater resolution in our understanding of ILC hematopoiesis. Here, we have combined the analysis of real-time in vivo transcription factor expression, using existing and novel polychromILC mice, with the expression of NKG2A/C/E cell surface markers to identify Lin^−^Id2^+^IL-7Rα^+^CD25^−^α4β7^−^NKG2A/C/E^+^Bcl11b^−^T-bet^+^Eomes^−^PLZF^lo/int^ transILCPs that are wholly restricted to the ILC1/NK cell lineage. We refer to these cells as “aceNKPs” (SI Appendix, Fig. S8).

In vitro these aceNKPs develop into innate lymphocytes that express NK1.1, NKp46, Eomes, and T-bet, as well as perforin and IFN-γ following IL-2, IL-15, and IL-18 stimulation. This ILC1/NK cell fate was confirmed in vivo with adoptive transfer of these aceNKPs giving rise exclusively to a spectrum of helper-like and cytotoxic-like ILC1/NK cells, regardless of the tissue-specific microenvironmental cues they encountered. Furthermore, our experiments using the B16F10 melanoma metastatic model demonstrate that aceNKPs give rise to fully functional antitumoral NK cells.

Following the discovery of additional ILC populations, reports suggested an early bifurcation of NKPs prior to the commitment of “helper” ILC progenitors (CHILPs) (11, 26, 50). These were further characterized by their high PLZF expression (26, 50, 69). However, more recently, using mice engineered to express *Id2-*RFP and *Zbtb16*-GFP, it was shown that CHILPs still retain NK cell (granzyme and perforin-producing) potential (28). Furthermore, the analysis of mice expressing Id2-BFP, Gata3-hCD2, Rorc-Katushka, Rora-Teal, and *Bcl11b*-tdTomato (27), in combination with scRNAseq, confirmed the contemporaneous development of “helper” ILCs (ILC1s, ILC2s, and ILC3s) and NK cells from a common transitional ILC progenitor population (transILCP) that could be further subdivided by their transcription factor reporter expression. TransILCP commitment was determined by Gata3 expression, with Gata3^high^ cells giving rise almost exclusively to the ILC2 lineage and Gata3^low^ cells retaining the capacity to produce all the ILC lineages (ILC1/NK cells, ILC2, and ILC3) (27). These results were confirmed when a refined CHILP isolation strategy indicated that previously described CHILPs still retained NK cell potential (30), and also clarified several early observations where ILC1-like cells derived from CHILPs expressed Eomes i.e., were NK-like (69).

Together these results raised questions about the developmental progression of NK cell ontogeny and suggested that there was greater complexity than had previously been realized. Indeed, putative NK progenitors were first identified before the discovery of the other ILC-family members as cells residing within the Lin^−^NK1.1^−^CD49b^−^CD122^+^ bone marrow hematopoietic cell compartment (32, 70). Later on, two concomitant reports further refined the identification of NKPs using CD27 and CD244, or Id2/Sca1 and c-Kit expression (21, 23). However, the discovery of ILCs and the use of fate mapping approaches indicated that the previously defined NKP populations could also contain ILC1, ILC2, and ILC3 progenitors (50). Consequently, although there is reasonable harmonization in our understanding of the immature and mature NK cell differentiation and restriction pathway (3, 6, 24), the critical stages in ILCP-NKP commitment have yet to be adequately defined.

Using the resolution provided by the detailed mapping of ILC development-regulating transcription factors allied with previously characterized cell surface marker expression has allowed us to define the aceNKP that is solely committed to the production of mature ILC1/NK cells, and lacks any remaining ILC progenitor capacity. When we assessed the lineage specification of previously defined pre-NKP and/or rNKP populations, we determined that they still retained the capability to produce ILC2 and ILC3, in line with a previous report indicating that pre-NKPs are comprised of a mixture of progenitors for NK cells and ILCs (50). Indeed, while the original reports showed that those progenitor cells that developed in vitro became NK cells, there was a fraction of pre-NKPs/rNKPs that failed generate NK cells (21, 23). Our results indicate that pre-NKPs/rNKPs contained a mixed population of progenitors displaying varying degrees of Id2, Bcl11b, CD25 and NKG2A/C/E expression, which would explain their residual ILC potential. In vivo these pre-NKP/rNKPs gave rise to ILCs when they populated the lung and the intestinal lamina propria, but, similar to what was observed in the original report defining rNKPs (23), ILC1/NK cells developed when pre-NKPs/rNKPs colonized the liver. In vitro the ILC potential of pre-NKPs, as reported previously, was likely masked by the inclusion of exogenous IL-15 (a potent differentiation factor for NK cells) in the differentiation cultures (21, 23, 71, 72). aceNKPs gave rise only to ILC1/NK cells in all the tissues analyzed, including the intestines, and exclusively produced ILC1/NK cells in vitro, even without the addition of IL-15. Indeed, stimulation of aceNKP-progeny with IL-15 pushed their development toward mature Eomes-, perforin-, and IFN-γ-positive NK-like cells. While we cannot rule out that our sorting strategy was slightly different from those used originally to define pre-NKPs and rNKPs (e.g., rNKP were described to express *Id2* transcripts but were not tested using Id2-reporter mice) (21, 23), we were able to recapitulate the commitment of the formerly described NKPs toward the NK lineage upon in vitro IL-15 supplementation and their in vivo NK development in the liver and spleen.

Historically, the expression of NKG2A/C/E was proposed to mark maturation stages within IL-7Rα-negative iNKs, but this was before the identification of rNKPs and ILCPs (60). Consequently, the expression of NKG2A/C/E in NKPs and ILCPs has not been explored in detail. Indeed, we now know that NKG2A/C/E molecules are not only expressed by NK cells but also by liver ILC1 and lamina propria ILC1/3s (54), and as shown here NKG2A/C/E identify the earliest IL-7Rα-positive ILC1/NK-committed transILCP prior to their down-regulation of IL-7Rα expression upon transition to iNKs. Interestingly, early efforts to recapitulate NK ontogeny in vitro and in vivo highlighted that CD94 and/or NKG2A expression was present to certain extents in NK1.1^−^CD122^+^ NK progenitors, supporting our observations (61, 70). Although we were able to detect ILC1-like cells within the progeny of aceNKPs, the discrimination of these cells from NK cells was inconclusive, in particular in the absence of IL-15-derived cues. This observation fits with recent evidence that transcriptional variation within type-1 ILCs can be influenced by multiple factors within tissue microenvironments, and partially supporting a model where ILC1 and NK cells are plastic transposable states of the same ILC lineage, which constitute a spectrum of ILC1/NK cell states, rather than sharply distinguishable subsets (52, 62–64). Our results support the proposition that aceNKPs are a homogeneous population producing aceNKP-progeny, which fall within the NK/ILC1 spectrum (finely tuned by the interplay between Hobit, Eomes, Tcf1, T-bet, and other factors). However, due to the inability to perform clonal in vivo reconstitution analysis, we cannot completely exclude the presence of currently undetectable heterogeneity within the aceNKP population, and that certain aceNKPs may be comparatively more skewed toward an ILC1- or NK cell-like fate.

Notably, the transcriptional signature, phenotype, and function of ILCs is inherently influenced by their tissue microenvironment (73). ILC1s and NK cells appear to display shifting phenotypes depending on their tissue residence/tropism. Recently, several reports have attempted to define signatures that distinguish NK cells from their ILC1 counterparts (12, 52, 53, 67). The differential expression of Eomes by NK cells and Hobit by ILC1s has been proposed to discriminate these cell types regardless of tissue imprinting (52). However, even ILC1s marked using Hobit translational fate-mapping mice were capable of producing cytotoxic activity, thus indicating the fluid nature of type-1 ILC biology (12, 67). Indeed, it was also shown recently that while Hobit is seemingly not required for NK cell development or function, NK cells up-regulate Hobit during MCMV infection (67). These observations further support the inherently plastic nature of ILC1 and NK cells in different contexts, and suggests that ILC1 and NK cells may actually represent a phenotypic spectrum of the same cell type that cannot be easily distinguished and can arise from a common committed progenitor, as indicated by our results. Surprisingly, by employing new T-bet-hCD4-DTR reporter mice, we were able to detect T-bet expression in aceNKPs, even though some reports have indicated that Eomes precedes T-bet expression in NK progenitors (14, 35, 44) and suggests greater complexity in T-bet expression, even though T-bet is not essential for NKP development (4, 22, 43, 74). This observation is likely due to the reporter providing greater sensitivity than intracellular antibody staining. In addition, NKPs in the embryonic thymus also express higher levels of *Tbx21* mRNA than *Eomes* mRNA, alongside the genes encoding for NKG2A/C/E (15), suggesting that the expression of NKG2A/C/E marks the point where transILCPs lose the ability to develop into ILC2 and ILC3, committing to a type 1 innate lymphocyte lineage.

In the future, it would be interesting to investigate the requirements for the development of aceNKPs. It is tempting to hypothesize that NKG2A is involved in the ontogeny of aceNKPs. However, it seems that this receptor is required for the education of NK cells rather than their development, suggesting that the NKG2A/C/E and CD94 receptors serve as identification markers, and aceNKPs do not depend on them for their development (55–57). While Nfil3 and Id2 are likely necessary for aceNKPs (33–36), Eomes and T-bet may not be required until aceNKPs progress to mature NK cells, based on previous findings (14, 43). Additionally, the study of the influence of Bcl11b and Hobit in promoting a more ILC1- or NK-like phenotype could help understanding how aceNKPs differentiate to contribute to the ILC1/NK cell continuum in tissues (12).

Interestingly, we observed that a subset of NKG2A/C/E^+^ tran-sILCPs expressed Bcl11b, which is known to be required for ILC development but is unnecessary for NK cell development (18, 75–78). It is important to note that the NKG2A/C/E^+^ Bcl11b^+^ transILCPs displayed lower levels of *Bcl11b*-tdTomato than the Bcl11b-positive NKG2A/C/E^−^ ILCPs/transILCPs, suggesting that subtle variations in Bcl11b expression may impact cell fate decisions. Based on their acquisition of NK1.1, NKp46, CD49a, and IFN-γ expression, in combination with their reduced production of perforin and Ly49 molecules, this NKG2A/C/E^+^Bcl11b^+^ population has characteristics suggestive of an ILC1P. However, in vivo only ~20% of the NKG2A/C/E^+^Bcl11b^+^ tran-sILCPs population became NK1.1^+^NKp46^+^ cells, raising the possibility that the NKG2A/C/E^+^Bcl11b^+^ population is an uncommitted transitional state that may require additional bone marrow-derived signals to support their commitment and proliferation (79). Indeed, *Bcl11b* mRNA has been detected in ILC1-committed progenitors in the liver, supporting our findings (12). Bcl11b expression by this putative ILC1P is a feature in common with ILC2Ps and ILC3Ps (18), suggesting a relationship characteristic of CHILP. Although CHILP are not adequately defined by PLZF or α4β7 expression (11, 26–28, 30, 50) our data suggest that a Bcl11b^+^ CHILP may produce ILC1P, ILC2P, and ILC3P progeny, a finding that warrants further investigation.

Together our results indicate that Lin^−^Id2^+^IL-7Rα^+^CD25^−^α4β7^−^NKG2A/C/E^+^Bcl11b^−^T-bet^+^Eomes^−^PLZF^lo/int^ transILCPs represent the earliest progenitor exclusively committed to the ILC1/NK lineage. We have called these progenitors aceNKPs to discriminate them from the previously reported pre-NKPs/rNKP population, that in our hands retains ILC potential. We suggest that a putative ILC1P may be contained within the Bcl11b-positive Lin^−^Id2^+^IL-7Rα^+^CD25^−^α4β7^−^NKG2A/C/E^+^ transILCP population, but further investigation is required. AceNKPs were able to fully mature into cytotoxic NK cells that confer protection against cancer metastasis.

## Materials and Methods

### Animals

All mice were bred in a specific pathogen-free facility. In individual experiments, mice were matched for age, sex, and background strain, and all experiments undertaken in this study were done so with the approval of the UK Home Office. *Eomes*-GFP mice were a gift from Elizabeth Robertson (Dunn School of Pathology, Oxford, UK) (80). *Bcl11b*-tdTomato mice were provided by Pentao Liu (Li Ka Shing Faculty of Medicine, Stem Cell and Regenerative Medicine Centre, University of Hong Kong, Hong Kong, China), and CD45.1 *Rag2*^–/–^*Il2rg*^–/–^ mice were a gift from James Di Santo (Institut Pasteur, Paris, France). C57BL/6JOla (referred to as WT in the figures) and transgenic mice were maintained in house. All other mouse strains had been previously defined (15, 27) or generated in the current project as indicated below.

### Generation of T-bet-hCD4 Gene-Targeted Mice

The *Tbx21* gene was targeted using CRISPR technology. Expression constructs encoding two guide RNAs of opposing orientation (G1 and G2) and the D10A nickase mutant of Cas9 were cotransfected into JM8 ES cells with the targeting construct shown in SI Appendix, Fig. S2A, to mediate insertion of the cassette by homology directed repair. The hCD4 coding region had a 33 amino acid C-terminal truncation (only leaving five amino acid of the intracellular region) to prevent intracellular signaling, and a mutation in the extracellular domain to preclude potential interactions with class II MHC molecules (81). Appropriate insertion was confirmed at the 3’ end by Southern blot analysis using the probe indicated on SI Appendix, Fig. S2A.

### Generation of *Zbtb16*-Citrine Gene-Targeted Mice

The *Zbtb16* gene was targeted using CRISPR technology. Expression constructs encoding two guide RNAs of opposing orientation (G1 and G2) and the D10A nickase mutant of Cas9 were cotransfected into JM8 ES cells with the targeting construct shown in SI Appendix, Fig. S3A, to mediate insertion of the cassette by homology directed repair. Appropriate insertion was confirmed at the 3’ end by Southern blot analysis using the probe indicated on SI Appendix, Fig. S3A.

### Data and Statistical Analyses

Statistical analysis was performed using GraphPad Prism v7.0b software.

The heatmap (Fig. 1A) was plotted in R using heatmap.2 (gplots version 3.0.1.1) with NK/ILC1-related genes from single-cell RNA sequencing data published previously: GSE131038 (27). Raw single-cell sequencing data generated in this study have been deposited in the NCBI Gene Expression Omnibus under accession number GSE213814.

## Supplementary Material

Appendix 01

## Data Availability

The single-cell RNA sequencing data have been deposited in GEO/NCBI (GSE213814). Some study data available (Data from this study, including flow cytometry data and material generated in this study such as mouse strains will be maintained at the MRC Laboratory of Molecular Biology and shared upon request. We have included and displayed all the data generated in this study or representative data where appropriate. Flow cytometry data has not been uploaded to a repository due to the size of the raw files, but it can be shared upon request without restriction.). Previously published data were used for this work (The data used to generated Figure 1A has been published by our group in Immunity journal on 2019. The publication where this data is originally published is cited and can be found with the GEO accession number GSE131038.).

## Significance

Changes in the balance of innate lymphoid cells (ILCs) can critically push anticancer immunity (mediated by natural killer (NK) cells) toward a tumor-permissive immune microenvironment (ILC2 or ILC3-mediated). ILCs develop from common ILC progenitors, but we and others have found previously defined NK cell-restricted progenitors (NKPs) still retain the ability to develop into other ILC lineages subject to tissue-specific cues. We delineate the existence of “aceNKPs” in murine bone marrow: NKG2A/C/E-expressing NKPs are the earliest hematopoietic precursors uniquely committed to become ILC1/NK cells with the capacity to further mature into group-1 ILCs capable of controlling tumor growth. Identification of aceNKPs is important for our understanding of the production of ILC1/NK cells and how they may be manipulated prior to maturation.
