# Supplementary material for: Identification of aceNKPs, a committed common progenitor population of the ILC1 and NK cell continuum
Source: Proc Natl Acad Sci U S A. 2022 Nov 29;119(49):e2203454119. doi: 10.1073/pnas.2203454119 (PMC7614094; doi:10.1073/pnas.2203454119)
Supplement: Supplementary file 1 — Appendix 01 (PDF) [file pnas.2203454119.sapp.pdf]

PNAS

Supporting Information for

Identification of aceNKPs, a committed common progenitor population of the ILC1 and NK cell continuum

Noe Rodriguez-Rodriguez[a,*], Paula A. Clark[a,1], Mayuri Gogoi[a,1], Ana C.F. Ferreira[a], Bernhard Kerscher[b], Alastair Crisp[a], Helen E. Jolin[a], Jane E. Murphy[a], Meera Sivasubramaniam[a], Luisa Pedro[c], Jennifer A. Walker[a], Morgan W.D. Heycock[a], Jacqueline D. Shields[c], Jillian L. Barlow[a,d,2,*] and Andrew N.J. McKenzie[a,2,*]

[a] MRC Laboratory of Molecular Biology, Cambridge, CB2 0QH, United Kingdom

[b] Paul-Ehrlich-Institute, Federal Institute for Vaccines and Biomedicines, Langen, Germany

[c] Hutchison/MRC Research Centre, Cambridge, CB2 0XZ, United Kingdom

[d] Present address: York Biomedical Research Institute, University of York, Department of Biology, Wentworth Way, YO10 5DD, United Kingdom

[1] These authors contributed equally to the manuscript

[2] Co-lead authors

*Corresponding authors: Noe Rodriguez-Rodriguez, Jillian L. Barlow, Andrew N.J. McKenzie

Email: noer@mrc-lmb.cam.ac.uk
jillian.barlow@york.ac.uk
anm@mrc-lmb.cam.ac.uk

This PDF file includes:

Supplementary Materials and Methods
Figures S1 to S8
SI References 82 to 84

Supplementary Materials and Methods.

Tissue preparation

Cell suspensions of spleen and liver were obtained by passing the tissues through a 70 μm strainer. Lung tissue and salivary glands were pre-digested by finely chopping the tissue and incubating it with 750 U/mL collagenase I (GIBCO) and 0.3 mg/mL DNaseI (Sigma-Aldrich) prior to obtaining a single cell suspension. Bone marrow was removed from femurs, tibiae and hips by centrifuging briefly at 5000 x g. For bone marrow, lung, liver and spleen cell suspensions, red blood cells were removed by incubation with RBC lysis solution (140 mM NH_4Cl, 17 mM Tris; pH 7.2). Lung lymphocytes were further enriched by centrifugation in 30% Percoll at 850 x g for 10 min (GE Healthcare), while liver lymphocytes were enriched in 40% Percoll at 780 x g for 20 min. For preparation of siLP lymphocytes, intestinal contents were removed by the application of gentle pressure along the length of the intestine. Intestines were opened longitudinally, cut into 3 cm long pieces and washed briefly by vortexing in PBS plus 10 mM HEPES (PBS/HEPES). Epithelial cells were removed by incubation with RPMI supplemented with 2% FCS, 1 mM dithiothreitol and 5 mM EDTA for 20 min at 37ºC with shaking (200 rpm) twice. Intestinal pieces were washed with PBS/HEPES and incubated, with shaking (200 rpm) at 37ºC with RPMI + 2% FCS, 0.125 KU/mL DNaseI (Sigma-Aldrich) and 62.5 mg/mL Liberase TL (Roche) until no large pieces of intestine remained. Cells were then passed through a 70 μm strainer, pelleted and separated over a 40%-80% gradient of Percoll at 600 x g for 20 min. siLP lymphocytes were isolated from the interface and prepared for flow cytometric analysis.

Flow cytometry

Single cell suspensions were incubated with fluorochrome-, or biotin-, conjugated antibodies in the presence of anti-CD16/CD32 antibody (Fc block, clone 2.4G2), followed by fluorochrome-conjugated streptavidin where necessary. Antibodies were from Biolegend (CD3e (145-2C11), CD11b (M1/70), IL-7Rα (SB/199), CD62L (MEL-14), c-Kit (2B8), CD90.2 (30-H12), Ly49C/F/I/H (14B1), TCRβ (H57-597), hCD2 (RPA-2.10), hCD4 (OKT4), NKG2A (16A11), NKG2D (CX5), ICOS (C398.4A), CD122 (TM-β1), CD94 (18d3), CD244 (m2B4(B6)458.1)), NKp46 (29A1.4), TRAIL (N2B2), CD49b (DX5), CXCR6 (SA051D1), Granzyme C (SFC1D8), c-Kit (2B8)); eBioscience (hCD2 (RPA-2.10), CD4 (GK1.5), CD5 (53-7.3), CD8α (53-6.7), CD19 (eBio1D3), NK1.1 (PK136), CD11c (N418),

Gr-1 (RB6-8C5), FcεRI (MAR-1), Ter-119 (TER-119), CD45 (30-F11), KLRG1 (2F1), NKp46 (29A1.4), CD49b (DX5), CD44 (IM7), α4β7 integrin (DATK32), Sca-1 (D7), CD25 (PC61.5), Flt3 (A2F10), Eomes (Dan11mag), Tbet (eBio4B10), perforin (S16009B), IFN-γ (XMG1.2), CD45.2 (104), CD27 (LG.7F9)); BD Biosciences (CD49a (Ha31/8), NKG2A/C/E (20d5), NK1.1 (PK136), CD11b (M1/70), hCD2 (RPA-2.10), Ly49D (4E5), Ly49H (3D10)); MD Bioproducts (ST2 (DJ8)), and Santa Cruz (PLZF (D-9)). 'Lineage' staining included antibodies specific for CD3, CD4, CD8α, CD11c, CD19, FcεRI, Gr-1, Ter-119 and TCRβ when spleen, lung, liver and lamina propria were analysed, and further included CD5, CD11b, CD49b, and NK1.1 when bone marrow was examined (although in some instances individual lineage markers were analysed in separate channels, as indicated in figures). All samples were co-stained with a cell viability dye (Fixable dye eFluor780, Invitrogen; or Zombie NIR, Biolegend) and analysis was performed on a 5-laser LSRFortessa system (BD Biosciences) or spectral cytometer ID7000 (Sony Biotechnology). For cell sorting an iCyt Synergy (70 mm nozzle, Sony Biotechnology) or FACSAria Fusion systems were used. Precision Count Beads (Biolegend) were used to calculate cell numbers. Intracellular transcription factor staining was performed by fixation with 2% PFA for 45 min at RT, followed by incubation with fluorochrome antibodies diluted in perm/wash buffer (Foxp3 staining kit, eBioscience). For the analysis of samples in Fig. 4, intracellular transcription factor staining was performed by fixation, followed by incubation with fluorochrome-conjugated antibodies using the Foxp3 staining kit (eBioscience). Intracellular cytokine was performed using BD Cytofix/Cytoperm Plus reagents (BD Biosciences) following culture with complete RPMI, supplemented with Cell Stimulation Cocktail or cytokines as indicated, plus protein transport inhibitors (eBioscience) for 4-10 h at 37ºC. Flow cytometric analysis, including unsupervised dimensionality reduction and clustering (82, 83), was performed using FlowJo, LLC v10 (BD) and associated plugins.

Single-cell RNA sequencing

Lungs were harvested and processed as indicated above prior to flow cytometry sorting of aceNKP-derived progeny as Live $CD45.2^+Id2^+$. Single-cell library preparation was performed using the 10x Genomics technology, and the 3′ libraries were obtained through the 10x Genomics Chromium Single-Cell 3′ v3 protocol and underwent subsequent sequencing. The reads were aligned to the mouse transcriptome (GRCm38), and expression and clusters were determined using 10x Genomics Cell Ranger (version

6.0.1). Further analysis and statistical calculations were performed using the 10x Genomics Loupe Browser (version 5.1) (https://support.10xgenomics.com/single-cell-gene-expression/software/visualization/latest/what-is-loupe-cell-browser). Data outliers such as contaminating myeloid cells and dead cells were removed from the analysis by applying the following parameters: UMI threshold: 500-20000; genes per barcode > 500; mitochondrial UMI < 5%; *Ptprc* expression > 0.1; *Sirpa*, *Cd63*, *Csf1r*, *Csf2ra*, *Trpm2*, *Clec9a* expression < 0.002.

Adoptive transfers

Populations of mouse bone marrow cells were FACS purified into heat-inactivated FCS, diluted to 50% with PBS and washed with PBS before injection. Cell suspensions were aspirated with a syringe and implanted via tail vein injection into sublethally-irradiated (450 rad) CD45.1 *Rag2*$^{-/-}$*Il2rg*$^{-/-}$ recipients. Analysis of donor cell progeny was performed 6 to 7 weeks after cell transfer.

OP9 stromal cell co-cultures

OP9 or OP9-DL4 cells were maintained in complete IMDM (IMDM, supplemented with 20% FCS, 1% penicillin, 1% streptomycin, 0.1% 2-mercaptoethanol and non-essential amino acids (GIBCO)). For progenitor cell co-culture, OP9 cells were incubated with 4 mg/mL mitomycin C for 2 h, thoroughly washed, seeded at a density of 1 x 10^6 cells per 96-well plate and allowed time to adhere. Sorted ILC progenitor populations were seeded onto OP9 monolayers and cultured in complete IMDM, supplemented with 25 ng/mL rmIL-7 (Biolegend) and 25 ng/mL rmSCF (Biolegend), for 7 days before flow cytometric analysis of progeny, unless otherwise indicated. For analysis of perforin, IFN-γ and Eomes (when indicated) expression cell subsets were seeded onto OP9 monolayers and cultured in complete IMDM, supplemented with 25 ng/mL rmIL-7 (Biolegend) and 25 ng/mL rmSCF (Biolegend), for 7 days and then stimulated with IL-2, IL-15 and IL-18 (all at 50 ng/mL) for a further 48 h before flow-cytometric analysis. Single cell clonal differentiation analysis was performed by directly sorting progenitors as single cell into individual wells with OP9 monolayers and culture them for 14-22 days with IL-7 and SCF. To analyse Eomes and T-bet expression by ICS on single cell clonal differentiation assays, cells were cultured with IL-2, IL-15 and IL-18 for further 48h. For analysis of B cell potential, cell subsets were seeded onto OP9 monolayers and cultured in complete IMDM, supplemented with 10 ng/mL rmIL-7, and 10 ng/mL rmFlt3L (Biolegend), for 13 days. For analysis of T cell

potential, cell subsets were seeded onto OP9-DL4 monolayers and cultured in complete IMDM, supplemented with 5 ng/mL rmIL-7, and 10 ng/mL rmFlt3L (Biolegend), for 24 days.

B16F10-mCherry melanoma cultures and tumour model

B16F10-mCherry (84) were grown in Dulbecco's Modified Eagle's Medium with 10% FCS 1% penicillin, and 1% streptomycin. Tumour cells were detached using 0.05% Trypsin-EDTA (Gibco) and washed twice in PBS before injection. 1 x 10^5 cells in PBS were transferred via tail vein injection. Metastatic foci/nodules in the left lung were quantified under the stereo-microscope by a blinded operator 14 days after intravenous administration.

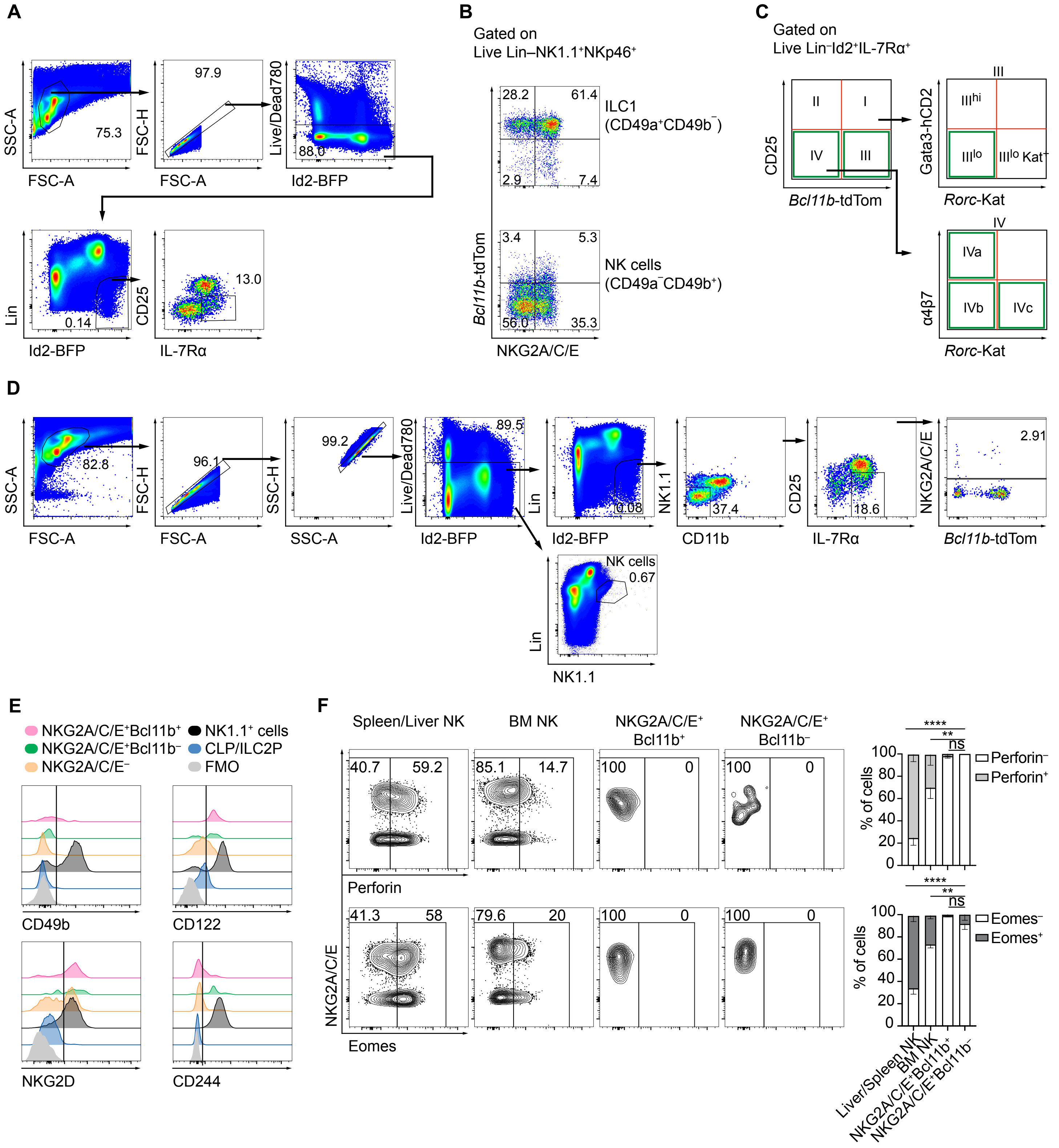
A
SSC-A
FSC-A
75.3
FSC-H
97.9
Live/Dead780
Id2-BFP
88.0
Lin
0.14
CD25
IL-7Rα
13.0
B
Gated on
Live Lin–NK1.1+NKp46+
28.2
61.4
2.9
7.4
ILC1
(CD49a+CD49b−)
3.4
5.3
56.0
35.3
NK cells
(CD49a−CD49b+)
Bcl11b-tdTom
NKG2A/C/E
C
Gated on
Live Lin−Id2+IL-7Rα+
II
I
IV
III
CD25
Bcl11b-tdTom
Gata3-hCD2
IIIhi
IIIlo
IIIlo Kat+
Rorc-Kat
IV
IVa
IVb
IVc
α4β7
Rorc-Kat
D
SSC-A
FSC-A
82.8
FSC-H
96.1
SSC-H
SSC-A
99.2
Live/Dead780
Id2-BFP
89.5
Lin
Id2-BFP
0.08
NK1.1
CD11b
37.4
CD25
IL-7Rα
18.6
NKG2A/C/E
Bcl11b-tdTom
2.91
NK cells
0.67
Lin
NK1.1
E
NKG2A/C/E+Bcl11b+
NKG2A/C/E+Bcl11b−
NKG2A/C/E−
NK1.1+ cells
CLP/ILC2P
FMO
CD49b
CD122
NKG2D
CD244
F
Spleen/Liver NK
BM NK
NKG2A/C/E+ Bcl11b+
NKG2A/C/E+ Bcl11b−
40.7
59.2
85.1
14.7
100
0
100
0
Perforin
41.3
58
79.6
20
100
0
100
0
NKG2A/C/E
Eomes
****
**
ns
Perforin−
Perforin+
% of cells
Eomes−
Eomes+
Liver/Spleen NK
BM NK
NKG2A/C/E+Bcl11b+
NKG2A/C/E+Bcl11b−

Figure S1. Related to Figure 1: Expression of CD94 and NKGA/C/E defines a sub-cluster of hematopoietic progenitors in the bone marrow that resemble type-1 ILC progenitors. **(A)** Gating strategy used to characterise $Lin^{-}Id2^{+}IL\text{-}7R\alpha^{+}CD25^{-}$ haematopoietic cells expressing CD94 or NKG2A/C/E. **(B)** Representative dot plots depicting the expression of *Bcl11b*-tdTomato and NKG2A/C/E on liver ILC1 and NK cells. Plots are representative of one out of three independent experiments (n = 6). **(C)** Schematic outline depicting the previously defined populations where ILCPs with ILC1/NK cell potential (highlighted in green) were identified by Walker and colleagues (2019). **(D)** Gating Strategy used to analyse the expression of NK cell-related markers in $NKG2A/C/E^{-}$, $NKG2A/C/E^{+}Bcl11b^{-}$, $NKG2A/C/E^{+}Bcl11b^{+}$ cells and NK cells. **(E)** Expression of CD49b (DX5, *Itga2*), CD122 (IL-2Rβ, *Il2rb*), NKG2D (*Klrk1*), and CD244 (2B4, *Cd244*) on several populations of bone marrow hematopoietic cells from 5x polychromILC reporter mice ($NKG2A/C/E^{-}$, $NKG2A/C/E^{+}Bcl11b^{-}$ and $NKG2A/C/E^{+}Bcl11b^{+}$ were pre-gated in Live $Lin^{-}Id2^{+}IL\text{-}7R\alpha^{+}CD25^{-}$ cells). Histograms are representative of two independent experiments (n = 11). **(F)** Expression of perforin and Eomes in steady-state splenic/hepatic NK cells, and bone marrow-resident $NKG2A/C/E^{+}Bcl11b^{-}$ and $NKG2A/C/E^{+}Bcl11b^{+}$ progenitors and NK cells from 4x polychromILC reporter mice (lacking $Gata3^{+/hCD2}$ reporter). Representative contour plots (left) and pooled cumulative data (right) from two independent experiments (n = 7 mice). Data presented as mean ± SEM. ns: no significant; ** $p < 0.01$; **** $p < 0.0001$; two-way ANOVA with Dunnett's multiple comparisons test.

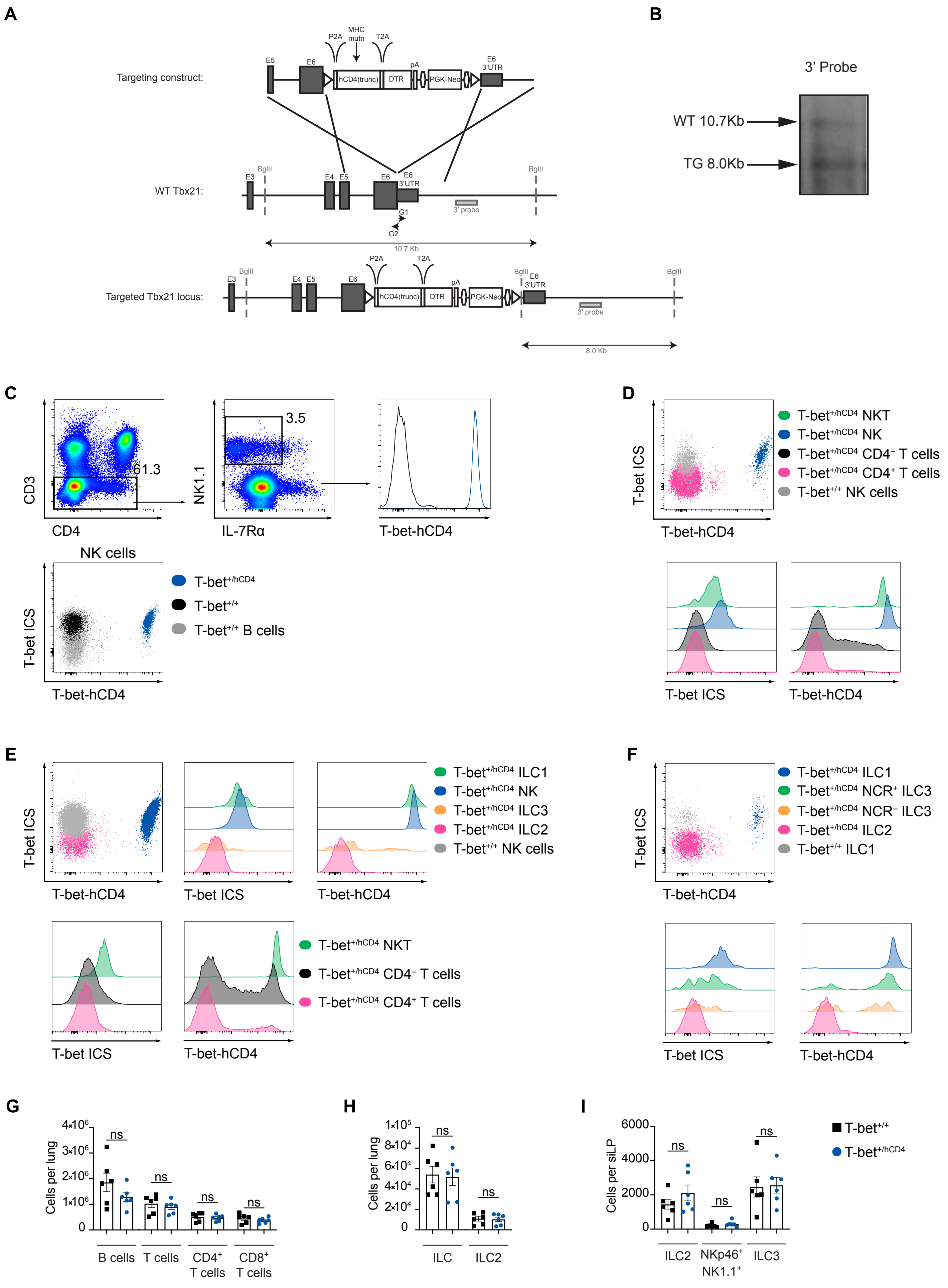

A
Targeting construct:
WT Tbx21:
Targeted Tbx21 locus:
P2A
MHC mutn
T2A
hCD4(trunc)
DTR
PGK-Neo
3' probe
10.7 Kb
8.0 Kb
B
3' Probe
WT 10.7Kb
TG 8.0Kb
C
CD3
CD4
61.3
NK1.1
IL-7Rα
3.5
T-bet-hCD4
NK cells
T-bet ICS
T-bet+/hCD4
T-bet+/+
T-bet+/+ B cells
D
T-bet+/hCD4 NKT
T-bet+/hCD4 NK
T-bet+/hCD4 CD4− T cells
T-bet+/hCD4 CD4+ T cells
T-bet+/+ NK cells
E
T-bet+/hCD4 ILC1
T-bet+/hCD4 NK
T-bet+/hCD4 ILC3
T-bet+/hCD4 ILC2
T-bet+/+ NK cells
F
T-bet+/hCD4 ILC1
T-bet+/hCD4 NCR+ ILC3
T-bet+/hCD4 NCR− ILC3
T-bet+/hCD4 ILC2
T-bet+/+ ILC1
G
Cells per lung
B cells
T cells
CD4+ T cells
CD8+ T cells
ns
H
ILC
ILC2
I
Cells per siLP
ILC2
NKp46+ NK1.1+
ILC3
T-bet+/+
T-bet+/hCD4

Figure S2. Related to Figure 1: T-bet-hCD4 mouse. (A) Gene-targeting strategy used to generate the T-bet-hCD4 (T-bet$^{+/hCD4}$) strain (WT = wildtype). **(B)** Southern blot analysis of *Bg*lII-digested genomic DNA from Tbx21-hCD4-DTR targeted ES cell clone using 3' probe indicated in (A). **(C)** T-bet-hCD4 expression in NK cells (CD45^{+}CD3^{-}IL-7Rα^{-}NK1.1^{+}) from the spleen of T-bet$^{+/hCD4}$ and WT littermates (T-bet$^{+/+}$) compared to intracellular staining (ICS) for T-bet. **(D-F)** T-bet-hCD4 expression and T-bet expression by ICS were evaluated in the indicated lymphocytic population from peripheral lymph nodes (D), lung (E) and small intestine (si) lamina propria (LP) cells from T-bet$^{+/hCD4}$ and/or WT littermates (T-bet$^{+/+}$). CD4^{+} T cells, CD45^{+}CD3^{+}CD4^{+}; CD4^{-} T cells, CD45^{+}CD3^{+}CD4^{-}; NK cells, CD45^{+}CD3^{-}IL-7Rα^{-}NK1.1^{+}; NKT cells, CD45^{+}CD3^{+}NK1.1^{+}; ILC1, CD45^{+}CD3^{-}Lin^{-}IL-7Rα^{+}NK1.1^{+}; ILC2, CD45^{+}CD3^{-}Lin^{-}IL-7Rα^{+}NK1.1^{-}Gata3^{+}; ILC3, CD45^{+}CD3^{-}Lin^{-}IL-7Rα^{+}NK1.1^{-}RORγt^{+}; NCR^{+}ILC3, CD45^{+}CD3^{-}Lin^{-}IL-7Rα^{+}NK1.1^{-}RORγt^{+}NKp46^{+}; NCR^{-}ILC3, CD45^{+}CD3^{-}Lin^{-}IL-7Rα^{+}NK1.1^{-}RORγt^{+}NKp46^{-}. **(G, H)** Number of the indicated lung lymphocytes in T-bet$^{+/hCD4}$ and C57BL/6 controls (B cells = CD45^{+}CD3^{-}CD19^{+}; T cells = CD45^{+}CD3^{+}CD19^{-}; CD4^{+} T cells = CD45^{+}CD3^{+}CD4^{+}CD8^{-}; CD8^{+} T cells = CD45^{+}CD3^{+}CD8^{+}CD4^{-}; ILC = CD45^{+}Lin^{-}IL-7Rα^{+}; ILC2 = CD45^{+}Lin^{-}IL-7Rα^{+}ST2^{+}). **(I)** Number of the indicated lymphocytes in T-bet$^{+/hCD4}$ and C57BL/6 controls in siLP (ILC2 = CD45^{+}Lin^{-}IL-7Rα^{+}Gata3^{+}RORγt^{-}; NKp46^{+}NK1.1^{+} = CD45^{+}Lin^{-}IL-7Rα^{+}KLRG1^{-}NKp46^{+}NK1.1^{+}; ILC3 = CD45^{+}Lin^{-}IL-7Rα^{+}Gata3^{-}RORγt^{+}). Data are representative from three independent experiments (C-F, n = 5-7 mice) or cumulative data from one experiment (n = 6 mice/genotype) (G-I). Data represented as mean ± SEM. ns: no significant; unpaired two-sided Student's t test.

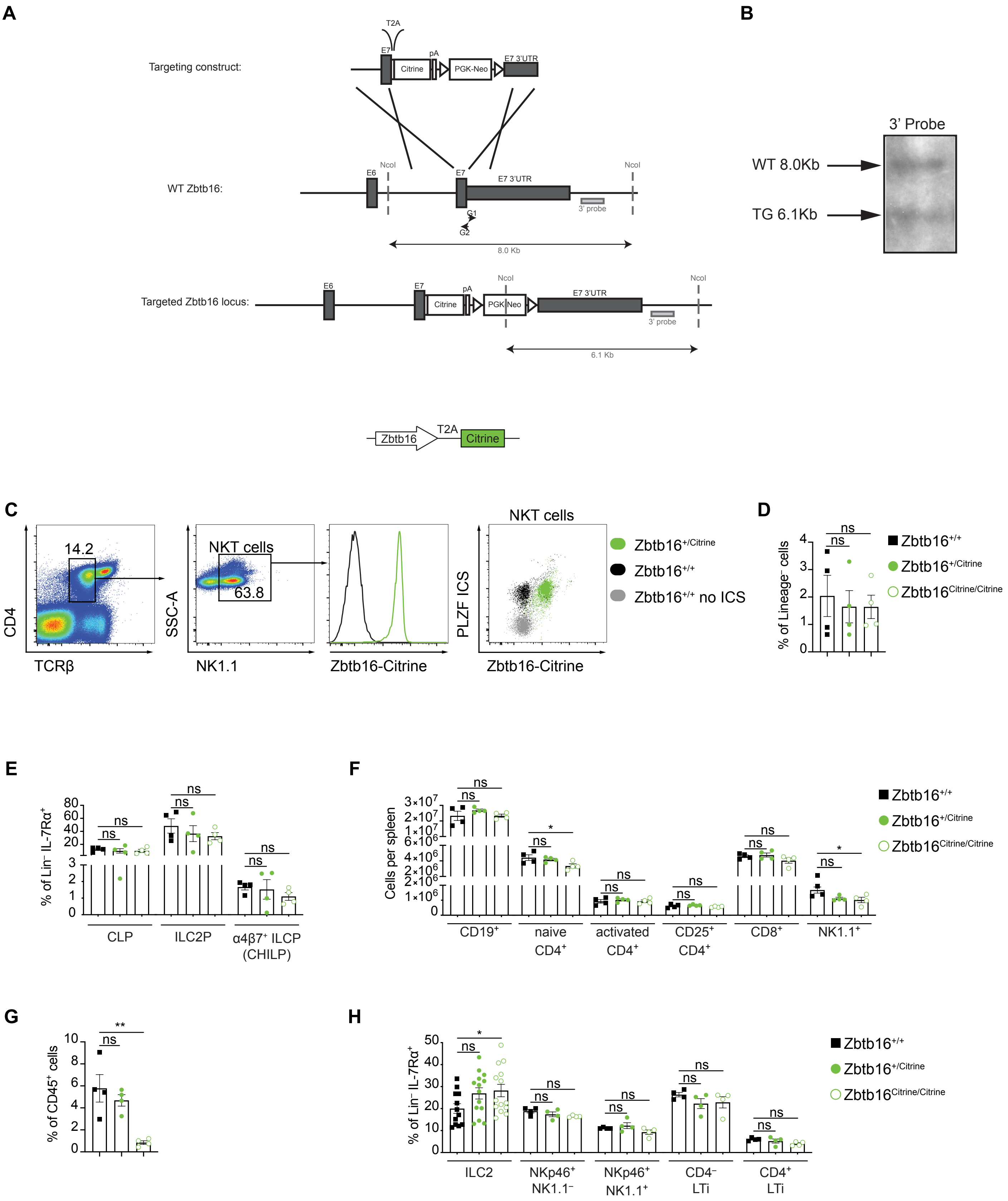

A
Targeting construct:
T2A
E7
pA
Citrine
PGK-Neo
E7 3'UTR
WT Zbtb16:
E6
NcoI
E7
E7 3'UTR
NcoI
3' probe
G1
G2
8.0 Kb
Targeted Zbtb16 locus:
E6
E7
pA
Citrine
PGK-Neo
NcoI
E7 3'UTR
NcoI
3' probe
6.1 Kb
Zbtb16
T2A
Citrine
B
3' Probe
WT 8.0Kb
TG 6.1Kb
C
14.2
CD4
TCRβ
NKT cells
63.8
SSC-A
NK1.1
Zbtb16-Citrine
NKT cells
PLZF ICS
Zbtb16-Citrine
Zbtb16+/Citrine
Zbtb16+/+
Zbtb16+/+ no ICS
D
% of Lineage− cells
ns
ns
Zbtb16+/+
Zbtb16+/Citrine
Zbtb16Citrine/Citrine
E
% of Lin− IL-7Rα+
CLP
ILC2P
α4β7+ ILCP (CHILP)
ns
F
Cells per spleen
CD19+
naive CD4+
activated CD4+
CD25+ CD4+
CD8+
NK1.1+
ns
*
Zbtb16+/+
Zbtb16+/Citrine
Zbtb16Citrine/Citrine
G
% of CD45+ cells
ns
**
H
% of Lin− IL-7Rα+
ILC2
NKp46+ NK1.1−
NKp46+ NK1.1+
CD4− LTi
CD4+ LTi
ns
*
Zbtb16+/+
Zbtb16+/Citrine
Zbtb16Citrine/Citrine

Figure S3. Related to Figure 1: Zbtb16-Citrine mouse. (A) Gene-targeting strategy used to generate the Zbtb16-Citrine (Zbtb16$^{+/Cit}$) strain. **(B)** Southern blot analysis of *Nco*I-digested genomic DNA from Zbtb16-Citrine targeted ES cell clone using 3' probe indicated in (A). **(C)** Zbtb16-Citrine in NKT cells (CD4^{+}TCRβ^{lo}NK1.1^{+}) from the liver of Zbtb16$^{+/Cit}$ and wildtype (WT) littermates (Zbtb16$^{+/+}$) compared to intracellular staining (ICS) for PLZF. **(D, E)** Frequency of HSCs (Lin$^{–}$CD45^{+}Kit^{+}Sca1^{+}Flt3$^{–}$) (D), and CLPs (Lin$^{–}$CD45^{+}IL-7Rα^{+}Flt3^{+}), ILC2Ps (Lin$^{–}$CD45^{+}IL-7Rα^{+}α4β7^{+}Sca1^{hi}Flt3$^{–}$CD25^{+}) and α4β7^{+} ILCPs (Lin$^{–}$CD45^{+}IL-7Rα^{+}α4β7^{hi}Sca1$^{int/lo}$ Flt3$^{–}$CD25$^{–}$; formerly denoted CHILP) (E) in bone marrow from Zbtb16$^{+/Cit}$ and Zbtb16$^{Ci/Cit}$ littermates and C57BL/6 controls. **(F)** Number of the indicated splenic lymphocytes in Zbtb16$^{+/Cit}$ and Zbtb16$^{Ci/Cit}$ littermates and C57BL/6 controls (naïve CD4^{+} = CD45^{+}CD3^{+}CD4^{+}CD62L^{hi}CD44^{lo}; activated CD4^{+} = CD45^{+}CD3^{+}CD4^{+}CD62L^{lo}CD44^{hi}; CD25^{+}CD4^{+} = CD45^{+}CD3^{+}CD4^{+}CD25^{+}; CD8^{+} = CD45^{+}CD3^{+}CD8^{+}; NK1.1^{+} = CD45^{+}CD3$^{–}$CD19$^{–}$NK1.1^{+}). **(G)** Frequency of NKT cells as defined in (C) in the liver of Zbtb16$^{+/Cit}$ and Zbtb16$^{Ci/Cit}$ littermates and C57BL/6 controls. **(H)** Frequency of the indicated ILCs from Zbtb16$^{+/Cit}$ and Zbtb16$^{Ci/Cit}$ littermates and C57BL/6 controls in siLP (ILC2 = CD45^{+}Lin$^{–}$IL-7Rα^{+} KLRG1^{+}; NKp46^{+}NK1.1$^{–}$ = CD45^{+}Lin$^{–}$IL-7Rα^{+}KLRG1$^{–}$CD4$^{–}$NKp46^{+}NK1.1$^{–}$; NKp46^{+}NK1.1^{+} = CD45^{+}Lin$^{–}$IL-7Rα^{+}KLRG1$^{–}$CD4$^{–}$ NKp46^{+}NK1.1^{+}; CD4$^{–}$LTi = CD45^{+}Lin$^{–}$IL-7Rα^{+}KLRG1$^{–}$CD4$^{–}$ NKp46$^{–}$NK1.1$^{–}$CCR6^{+}; CD4^{+}LTi = CD45^{+}Lin$^{–}$IL-7Rα^{+}KLRG1$^{–}$CD4^{+}CCR6^{+}). Data are from one experiment (n = 4 mice/genotype) (C-H), or pooled from four independent experiments (n = 12-14 mice/genotype) (ILC2 in H). Data represented as mean ± SEM. ns: no significant; * $p < 0.05$; ** $p < 0.01$; one-way ANOVA with Dunnett's multiple comparisons test.

A

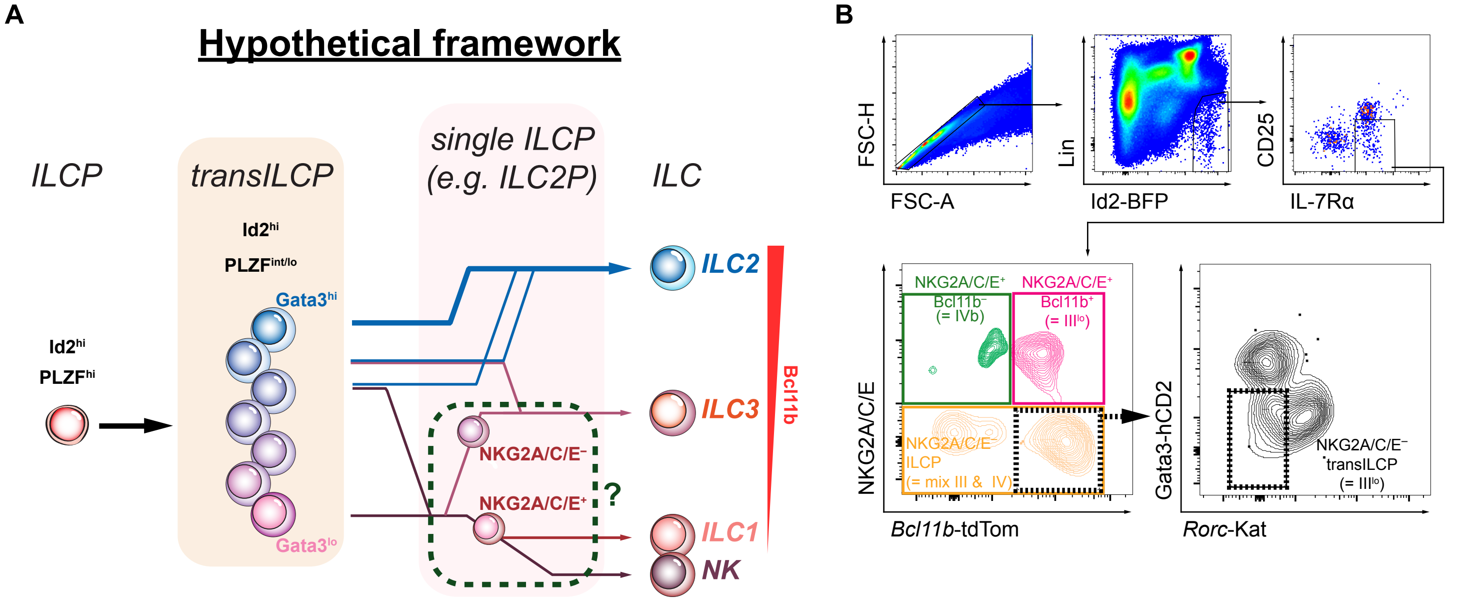

C

Population Name	Phenotype	Equivalent population in Walker et al, 2019
NKG2A/C/E⁻ ILCP	$CD45^{+}Lin^{-}Id2^{+}IL\text{-}7R\alpha^{+}CD25^{-}$ $Bcl11b^{-/+}Gata3^{-/+}\alpha4\beta7^{-/+}$ $ROR\gamma t^{-/+}ROR\alpha^{-/+}PLZF^{lo\text{-}hi}$ $T\text{-}bet^{-/+}NKG2A/C/E^{-}$	Mix of III & IV (NKG2A/C/E⁻ fraction)
NKG2A/C/E⁻ transILCP	$CD45^{+}Lin^{-}Id2^{+}IL\text{-}7R\alpha^{+}CD25^{-}$ $Bcl11b^{+}Gata3^{-}\alpha4\beta7^{-}$ $ROR\gamma t^{-}ROR\alpha^{-/+}PLZF^{int/hi}$ $T\text{-}bet^{-}NKG2A/C/E^{-}$	III^{lo} (NKG2A/C/E⁻ fraction)
NKG2A/C/E⁺Bcl11b⁺ transILCP	$CD45^{+}Lin^{-}Id2^{+}IL\text{-}7R\alpha^{+}CD25^{-}$ $Bcl11b^{+}Gata3^{-}\alpha4\beta7^{-}$ $ROR\gamma t^{-}ROR\alpha^{-}PLZF^{lo/int}$ $T\text{-}bet^{+}NKG2A/C/E^{+}$	III^{lo} (NKG2A/C/E⁺ fraction)
NKG2A/C/E⁺Bcl11b⁻ transILCP	$CD45^{+}Lin^{-}Id2^{+}IL\text{-}7R\alpha^{+}CD25^{-}$ $Bcl11b^{-}Gata3^{-}\alpha4\beta7^{-}$ $ROR\gamma t^{-}ROR\alpha^{-}PLZF^{lo/int}$ $T\text{-}bet^{+}NKG2A/C/E^{+}$	IVb (NKG2A/C/E⁺ fraction)

D

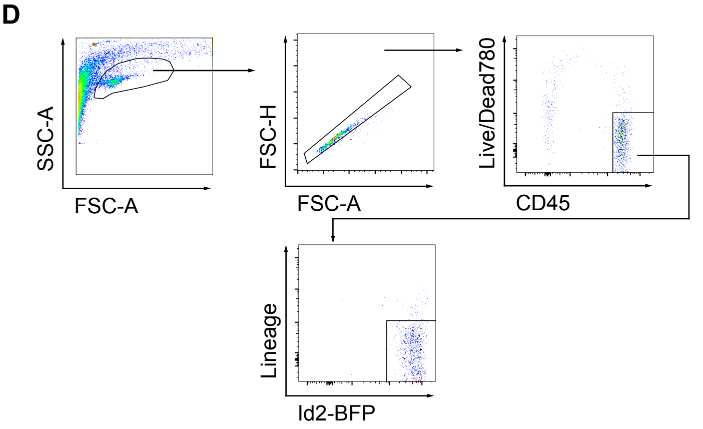

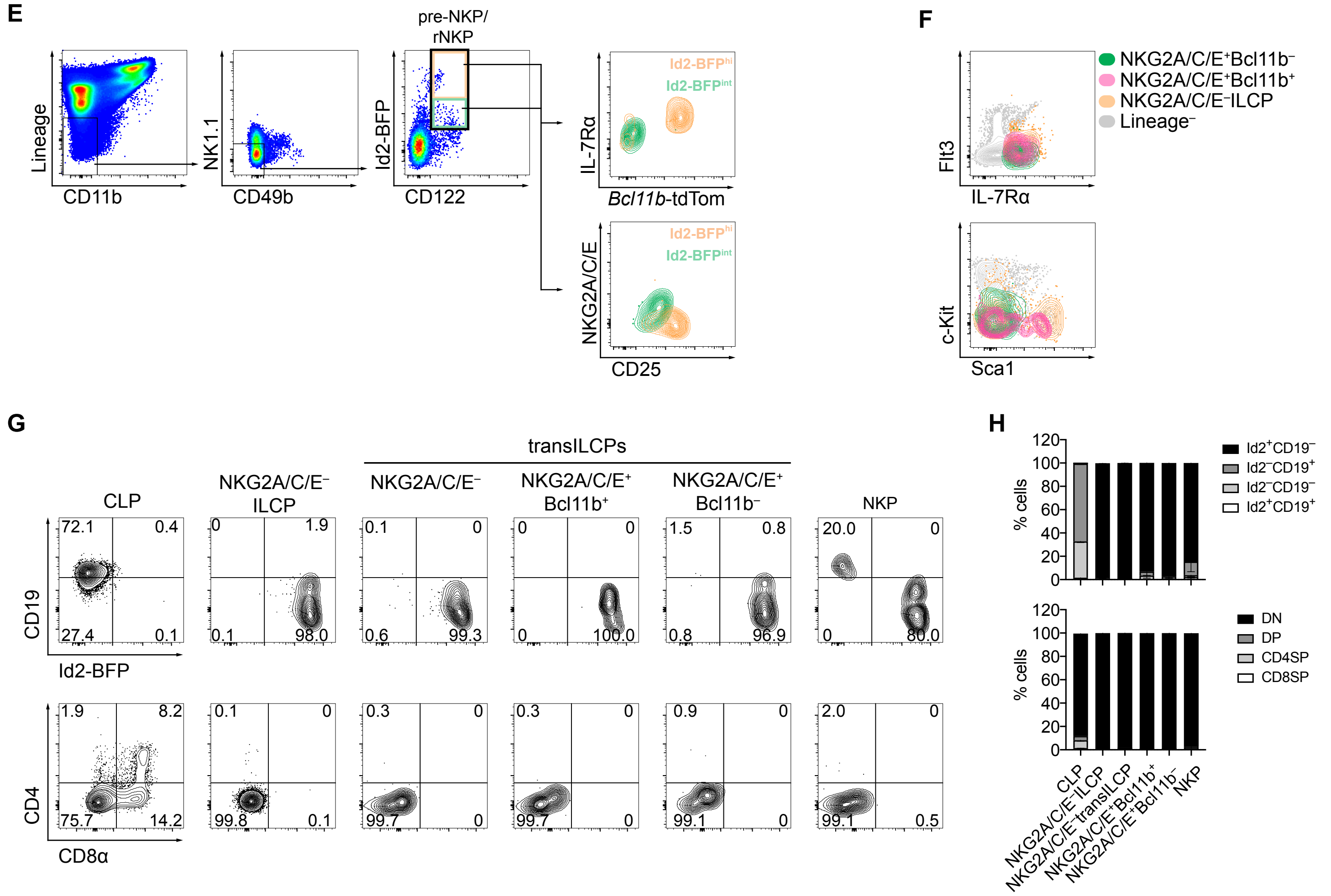

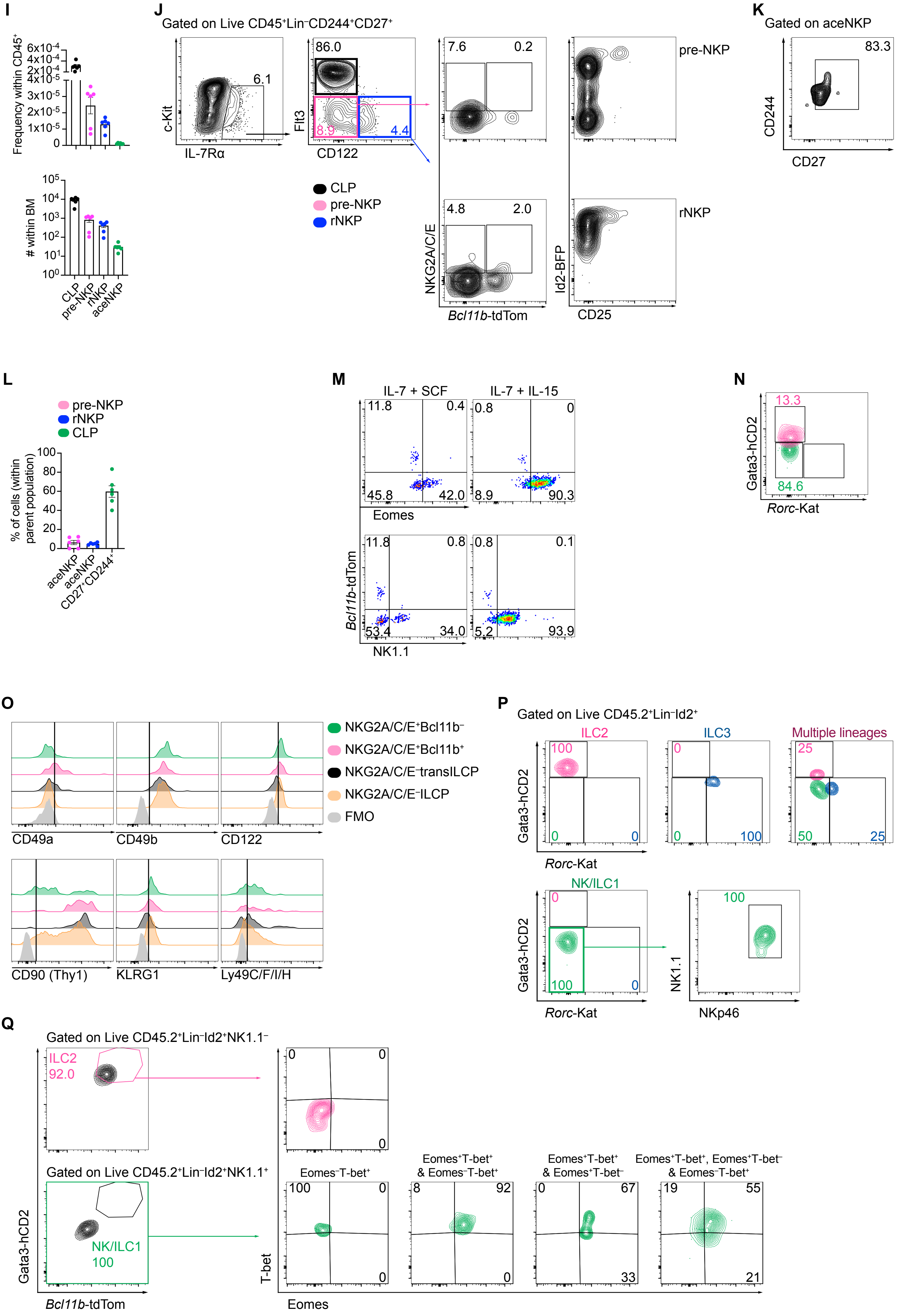
I
Frequency within CD45+
6x10-4
4x10-4
2x10-4
4x10-5
3x10-5
2x10-5
1x10-5
0
within BM
105
104
103
102
101
100
CLP
pre-NKP
rNKP
aceNKP
J
Gated on Live CD45+Lin-CD244+CD27+
c-Kit
IL-7Rα
6.1
Flt3
CD122
86.0
8.9
4.4
CLP
pre-NKP
rNKP
7.6
0.2
4.8
2.0
NKG2A/C/E
Bcl11b-tdTom
Id2-BFP
CD25
pre-NKP
rNKP
K
Gated on aceNKP
83.3
CD244
CD27
L
pre-NKP
rNKP
CLP
% of cells (within parent population)
100
80
60
40
20
0
aceNKP
aceNKP
CD27+CD244+
M
IL-7 + SCF
IL-7 + IL-15
11.8
0.4
45.8
42.0
0.8
0
8.9
90.3
Eomes
11.8
0.8
53.4
34.0
0.8
0.1
5.2
93.9
Bcl11b-tdTom
NK1.1
N
13.3
84.6
Gata3-hCD2
Rorc-Kat
O
NKG2A/C/E+Bcl11b−
NKG2A/C/E+Bcl11b+
NKG2A/C/E−transILCP
NKG2A/C/E−ILCP
FMO
CD49a
CD49b
CD122
CD90 (Thy1)
KLRG1
Ly49C/F/I/H
P
Gated on Live CD45.2+Lin−Id2+
ILC2
ILC3
Multiple lineages
100
0
0
0
0
100
25
50
25
Gata3-hCD2
Rorc-Kat
NK/ILC1
0
100
0
100
NK1.1
NKp46
Q
Gated on Live CD45.2+Lin-Id2+NK1.1−
ILC2
92.0
0
0
0
Gated on Live CD45.2+Lin-Id2+NK1.1+
NK/ILC1
100
Gata3-hCD2
Bcl11b-tdTom
Eomes−T-bet+
Eomes+T-bet+ & Eomes−T-bet+
Eomes+T-bet+ & Eomes+T-bet−
Eomes+T-bet+, Eomes+T-bet− & Eomes−T-bet+
100
0
0
8
92
0
0
67
33
19
55
21
T-bet
Eomes

Figure S4. Related to Figure 2: NKGA/C/E expression identifies hematopoietic progenitors exclusively committed to ILC1/NK lineage *in vitro*. **(A)** Ontogeny model used to test the hypothesis that NKG2A/C/E$^+$ cells may represent NK progenitors. **(B)** Gating strategy used to sort NKG2A/C/E$^-$ ILCPs, NKG2A/C/E$^-$ transILCPs, NKG2A/C/E$^+$Bcl11b$^+$ transILCPs, and NKG2A/C/E$^+$Bcl11b$^-$ transILCPs. When compared to the ILCP populations defined in our previous study (Walker et al, 2019), NKG2A/C/E$^-$ ILCPs are a mix of NKG2A/C/E$^-$ progenitors belonging to subsets III and IV (including $\alpha4\beta7^+$ ILCPs); NKG2A/C/E$^-$ transILCPs correspond to NKG2A/C/E$^-$ III^{lo} cells; NKG2A/C/E$^+$Bcl11b$^+$ transILCPs are equivalent to population NKG2A/C/E$^+$ III^{lo}, and NKG2A/C/E$^+$Bcl11b$^-$ transILCPs reside within population IVb. **(C)** Defining phenotype of NKG2A/C/E$^-$ ILCPs, NKG2A/C/E$^-$ transILCPs, NKG2A/C/E$^+$Bcl11b$^+$ transILCPs, and NKG2A/C/E$^+$Bcl11b$^-$ transILCPs and their equivalent populations as described in Walker et al, 2019. **(D)** Gating strategy applied to investigate the phenotype of the progeny from progenitors sorted in (B) prior to the contour plots shown in Fig. 2 A-D. **(E)** Gating strategy applied to identify pre-NKPs/rNKPs (NKP) in 5x polychromILC mice (black square). Pre-NKPs/rNKPs where further subdivided in Id2-BFP^{hi} (orange) and Id2-BFP^{int} (green) populations, and their expression of CD25, Bcl11b, IL-7Rα and NKG2A/C/E was analysed. **(F)** Representative contour plots analysing the expression of Flt3 (CD135), c-Kit (CD117) and Sca1 in NKG2A/C/E$^+$ and NKG2A/C/E$^-$ ILCP/transILCP populations. Contour plots are representative of two independent experiments ($n = 11$). **(G and H)** Representative contour plots (G) and cumulative data (H) of the differentiation of several lymphoid progenitors into B cells (CD19$^+$ cells; upper panels, after 13 days on OP9, IL-7, 10 ng/mL; Flt3L, 10 ng/mL) or T cells (CD4$^+$ or CD8$^+$ cells; lower panel, after 24 days on OP9-DL4 with IL-7, 5 ng/mL; and Flt3L, 10 ug/mL). CD4SP: CD4 single positive = CD4$^+$CD8$^-$; CD8SP: CD8 single positive = CD4$^-$CD8$^+$; DP: double positive = CD4$^+$CD8$^+$; DN: double negative = CD4$^-$CD8$^-$. Data representative (left) and pooled cumulative data from two independent experiment ($n = 5$). **(I)** Frequency (top) and total numbers (bottom) of CLPs, pre-NKPs, rNKPs and aceNKPs present in femora, tibiae, and ilia of 5x polychromILC reporter mice. Cumulative data from three independent experiments ($n = 6$). **(J)** Representative contour plots indicating the gating strategy to define pre-NKPs and rNKPS according to Fathman and colleagues (2011), and the expression of Id2-BFP, CD25, NKG2A/C/E and *Bcl11b*-tdTom. Numbers indicate percentage of cells within indicated gates. **(K)** Representative contour plot depicting CD244 and CD27 double-positive aceNKPs. **(L)** Cumulative data showing the proportion of aceNKP-like cells within pre-NKPs, rNKPs (as defined in J) or CD27$^+$CD244$^+$ cells within aceNKPs. Cumulative data from three independent experiments ($n = 6$). **(M)** Pre-NKPs/rNKPs were sorted and culture on OP9 cells with IL-7 and SCF or IL-7 and IL-15 for 7 days. Representative expression of *Bcl11b*-tdTomato, NK1.1 and Eomes in differentiated lymphocytes. Data

representative of one experiment (n = 2). **(N)** rNKPs as defined in (J) were sorted from bone marrow of 5x polychromILC reporter mice and culture on OP9 cells with IL-7 and SCF for 7 days. Representative expression of Gata3-hCD2, *Rorc*-Katushka in differentiated lymphocytes. **(O)** Cells from Fig. 2A were further cultured for 48h in the presence of IL-2, IL-15 and IL-18, and protein transport inhibitor for the last 4-6h. Expression of CD49a, CD49b, CD122, CD90 (Thy1), KLRG1, Ly49C/F/I/H (J) in Gata3^{-}RORγt^{-} cells. **(P and Q)** Examples of the results obtained from the single cell differentiation assays to define NK/ILC1, ILC2, and ILC3 (related to Fig 2J and 2K respectively). Numbers depicted in panels indicate frequency of cells. Representative data from two independent experiments (J, n = 11). Data presented as mean ± SEM.

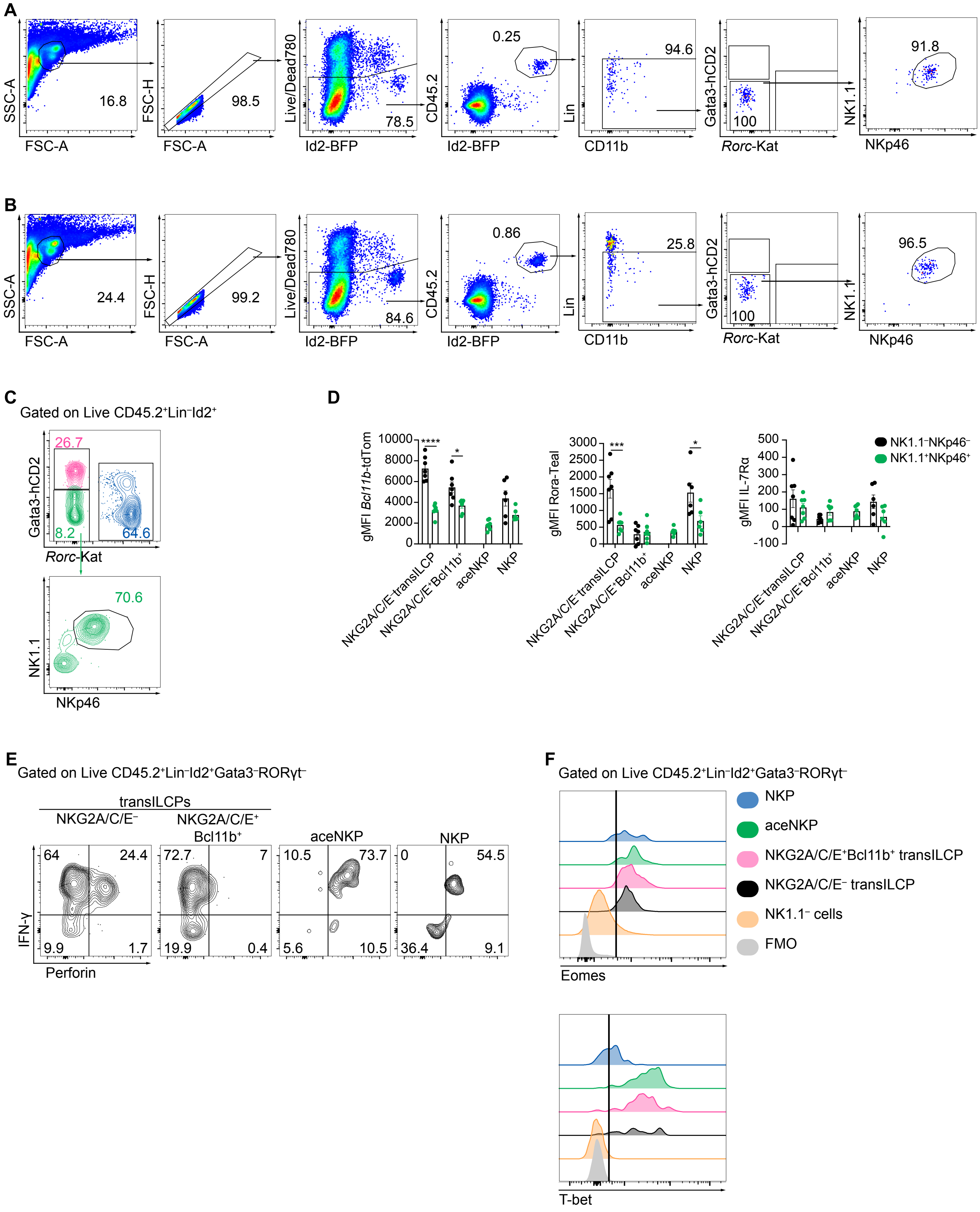
A
SSC-A
FSC-A
16.8
FSC-H
98.5
Live/Dead780
Id2-BFP
78.5
CD45.2
0.25
Lin
CD11b
94.6
Gata3-hCD2
Rorc-Kat
100
NK1.1
NKp46
91.8
B
24.4
99.2
84.6
0.86
25.8
96.5
C
Gated on Live CD45.2⁺Lin⁻Id2⁺
26.7
8.2
64.6
70.6
D
gMFI Bcl11b-tdTom
gMFI Rora-Teal
gMFI IL-7Rα
NK1.1⁻NKp46⁻
NK1.1⁺NKp46⁺
NKG2A/C/E⁻transILCP
NKG2A/C/E⁺Bcl11b⁺
aceNKP
NKP
E
Gated on Live CD45.2⁺Lin⁻Id2⁺Gata3⁻RORγt⁻
transILCPs
NKG2A/C/E⁻
NKG2A/C/E⁺ Bcl11b⁺
aceNKP
NKP
IFN-γ
Perforin
64
24.4
9.9
1.7
72.7
7
19.9
0.4
10.5
73.7
5.6
10.5
0
54.5
36.4
9.1
F
Gated on Live CD45.2⁺Lin⁻Id2⁺Gata3⁻RORγt⁻
NKP
aceNKP
NKG2A/C/E⁺Bcl11b⁺ transILCP
NKG2A/C/E⁻ transILCP
NK1.1⁻ cells
FMO
Eomes
T-bet

Figure S5. Related to Figure 3: aceNKP (NKG2A/C/E^{+}Bcl11b^{-} transILCP) progeny is restricted to ILC1/NK cells regardless of tissue microenvironment. (A) Gating strategy applied to investigate the phenotype of the progeny derived from the following sorted progenitor populations: NKG2A/C/E^{-} transILCPs, NKG2A/C/E^{+}Bcl11b^{+} transILCPs, aceNKPs, and NKPs. **(B)** Gating strategy depicting an example where lineage^{hi} progeny was detected in adoptive transfers, likely from contaminating CLPs due to the low frequency of sorted ILCP populations (6 out of 25 recipient mice). **(C)** Representative contour plots from siLP depicting proportions of Gata3^{+} (pink), RORγt^{+} (blue), and Gata3^{-}RORγt^{-} (green) lymphocytes (top) and proportions of NK cells (NK1.1^{+}NKp46^{+}) within Gata3^{-}RORγt^{-} (green) cells derived from derived from adoptively transferred rNKPs. **(D)** Cumulative data for expression of *Bcl11b*-tdTom, Rora-Teal and IL-7Rα in NK1.1^{+}NKp46^{+} and NK1.1^{-}NKp46^{-} within Gata3^{-}RORγt^{-} donor cells. **(E and F)** Representative dot plots indicating the frequencies of perforin- and IFN-γ-expressing cells (E), and Eomes and T-bet expression in cells (F) from liver samples in Fig. 3A after 24h of *in vitro* stimulation with IL-2, IL-15, and IL-18. Representative plots and pooled cumulative data from one (C, E and F, n = 3-4/group) or two independent experiments (D, n = 6-7 animal/group). Cumulative data represented as mean ± SEM. * $p < 0.05$; *** $p < 0.001$; **** $p < 0.0001$; two-way ANOVA with Dunnett's multiple comparisons test.

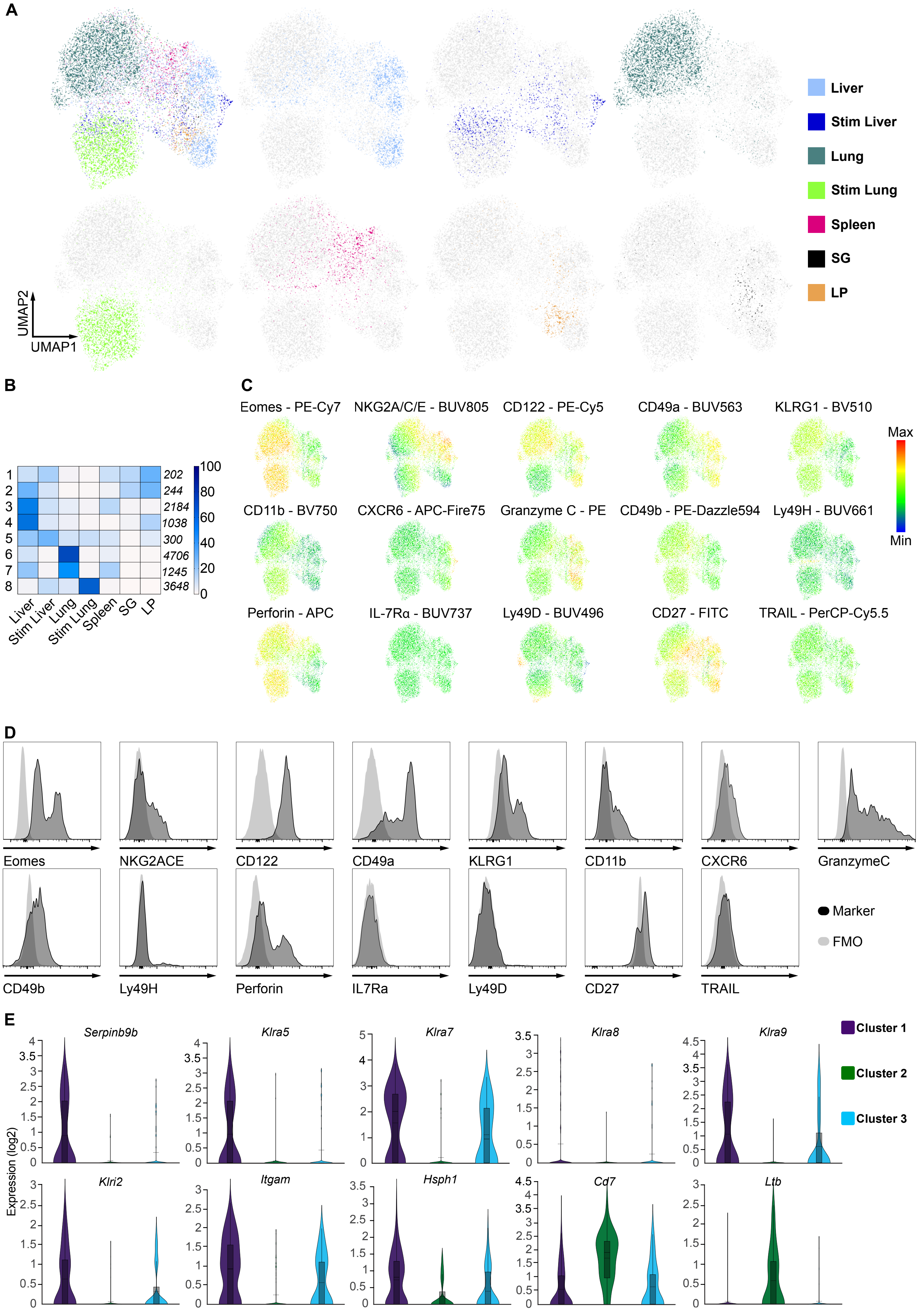
A
Liver
Stim Liver
Lung
Stim Lung
Spleen
SG
LP
UMAP2
UMAP1
B
1 202
2 244
3 2184
4 1038
5 300
6 4706
7 1245
8 3648
100
80
60
40
20
0
Liver
Stim Liver
Lung
Stim Lung
Spleen
SG
LP
C
Eomes - PE-Cy7
NKG2A/C/E - BUV805
CD122 - PE-Cy5
CD49a - BUV563
KLRG1 - BV510
CD11b - BV750
CXCR6 - APC-Fire75
Granzyme C - PE
CD49b - PE-Dazzle594
Ly49H - BUV661
Perforin - APC
IL-7Rα - BUV737
Ly49D - BUV496
CD27 - FITC
TRAIL - PerCP-Cy5.5
Max
Min
D
Eomes
NKG2ACE
CD122
CD49a
KLRG1
CD11b
CXCR6
GranzymeC
CD49b
Ly49H
Perforin
IL7Ra
Ly49D
CD27
TRAIL
Marker
FMO
E
Serpinb9b
Klra5
Klra7
Klra8
Klra9
Klri2
Itgam
Hsph1
Cd7
Ltb
Expression (log2)
Cluster 1
Cluster 2
Cluster 3

Figure S6. Related to Figure 4: aceNKPs represent a common progenitor of cytotoxic and helper ILC1/NK cells. (A) UMAP visualization of *in vivo* aceNKP-derived progeny coloured by tissue of origin. **(B)** Heatmap depicting the normalized contribution from each tissue to the cells comprising each identified cluster. Total cell numbers integrating each cluster are indicated on the right. **(C)** UMAP visualization of the indicated markers across the aceNKP-derived progeny coloured by gMFI. **(D)** Representative histograms showing the expression of the indicated markers on liver aceNKP-derived progeny (black) with their respective FMOs (gray). **(E)** Violin plots for genes differentially expressed across clusters 1-3 (as shown in Fig. 4D) identified from lung aceNKP-derived progeny. Pooled cumulative data from one (E = 3) or two/three independent experiments (A-D = 4-7 animal/tissue).

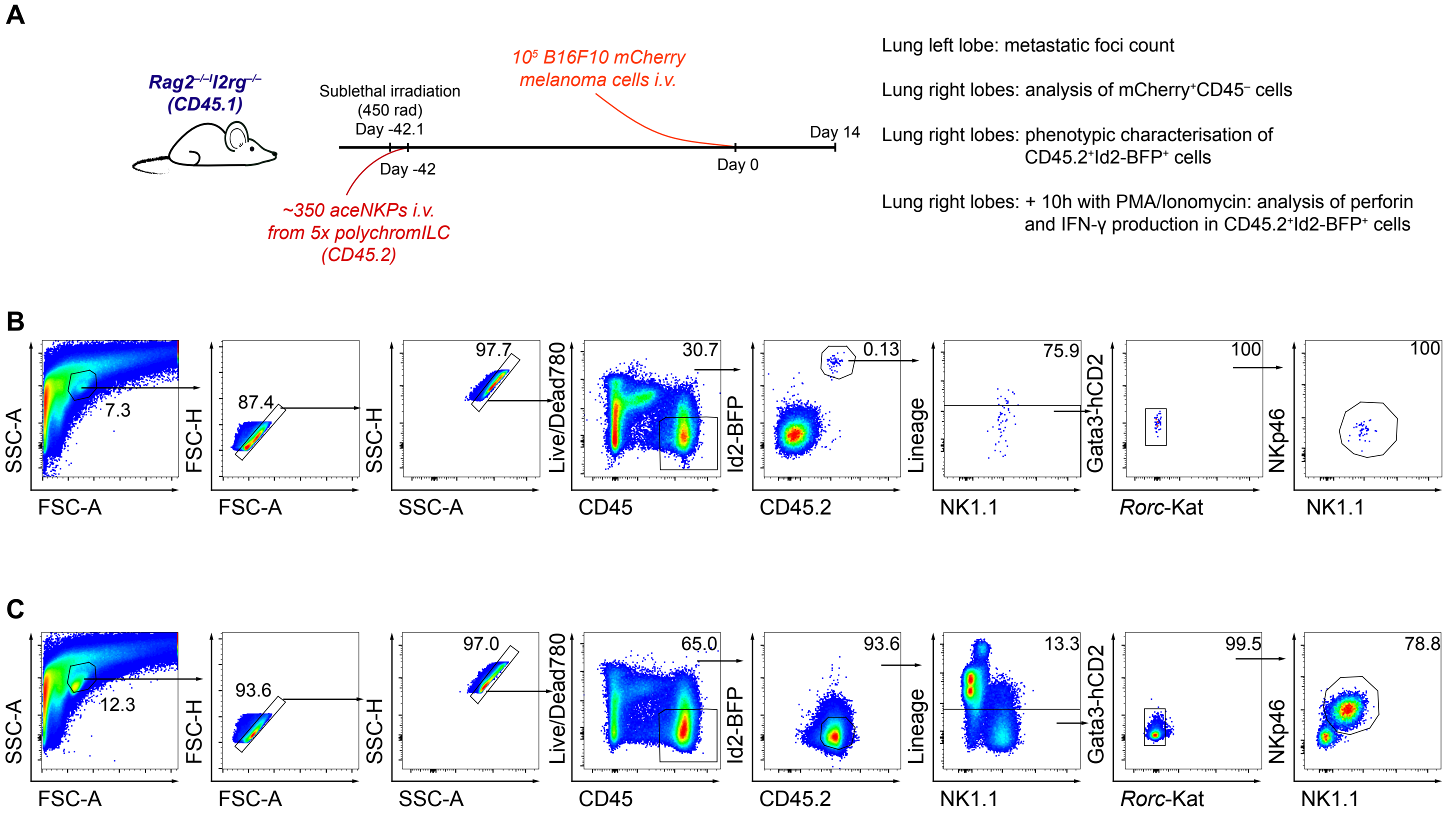
A
Rag2−/−Il2rg−/−
(CD45.1)
Sublethal irradiation
(450 rad)
Day -42.1
Day -42
~350 aceNKPs i.v.
from 5x polychromILC
(CD45.2)
10⁵ B16F10 mCherry
melanoma cells i.v.
Day 0
Day 14
Lung left lobe: metastatic foci count
Lung right lobes: analysis of mCherry⁺CD45⁻ cells
Lung right lobes: phenotypic characterisation of CD45.2⁺Id2-BFP⁺ cells
Lung right lobes: + 10h with PMA/Ionomycin: analysis of perforin and IFN-γ production in CD45.2⁺Id2-BFP⁺ cells
B
SSC-A
FSC-A
7.3
FSC-H
FSC-A
87.4
SSC-H
SSC-A
97.7
Live/Dead780
CD45
30.7
Id2-BFP
CD45.2
0.13
Lineage
NK1.1
75.9
Gata3-hCD2
Rorc-Kat
100
NKp46
NK1.1
100
C
SSC-A
FSC-A
12.3
FSC-H
FSC-A
93.6
SSC-H
SSC-A
97.0
Live/Dead780
CD45
65.0
Id2-BFP
CD45.2
93.6
Lineage
NK1.1
13.3
Gata3-hCD2
Rorc-Kat
99.5
NKp46
NK1.1
78.8

Figure S7. Related to Figure 5: aceNKP progeny present antitumor activity *in vivo*. (A) Schematic outline depicting the experimental design used in Fig. 5. **(B)** Gating strategy applied to investigate the phenotype of the progeny derived from aceNKPs in combination with the B16F10 metastatic tumour model. **(C)** Gating strategy applied to analyse the phenotype of NK cells residing in the lung of immunocompetent WT animals receiving intravenous injection of B16F10 tumour cells.

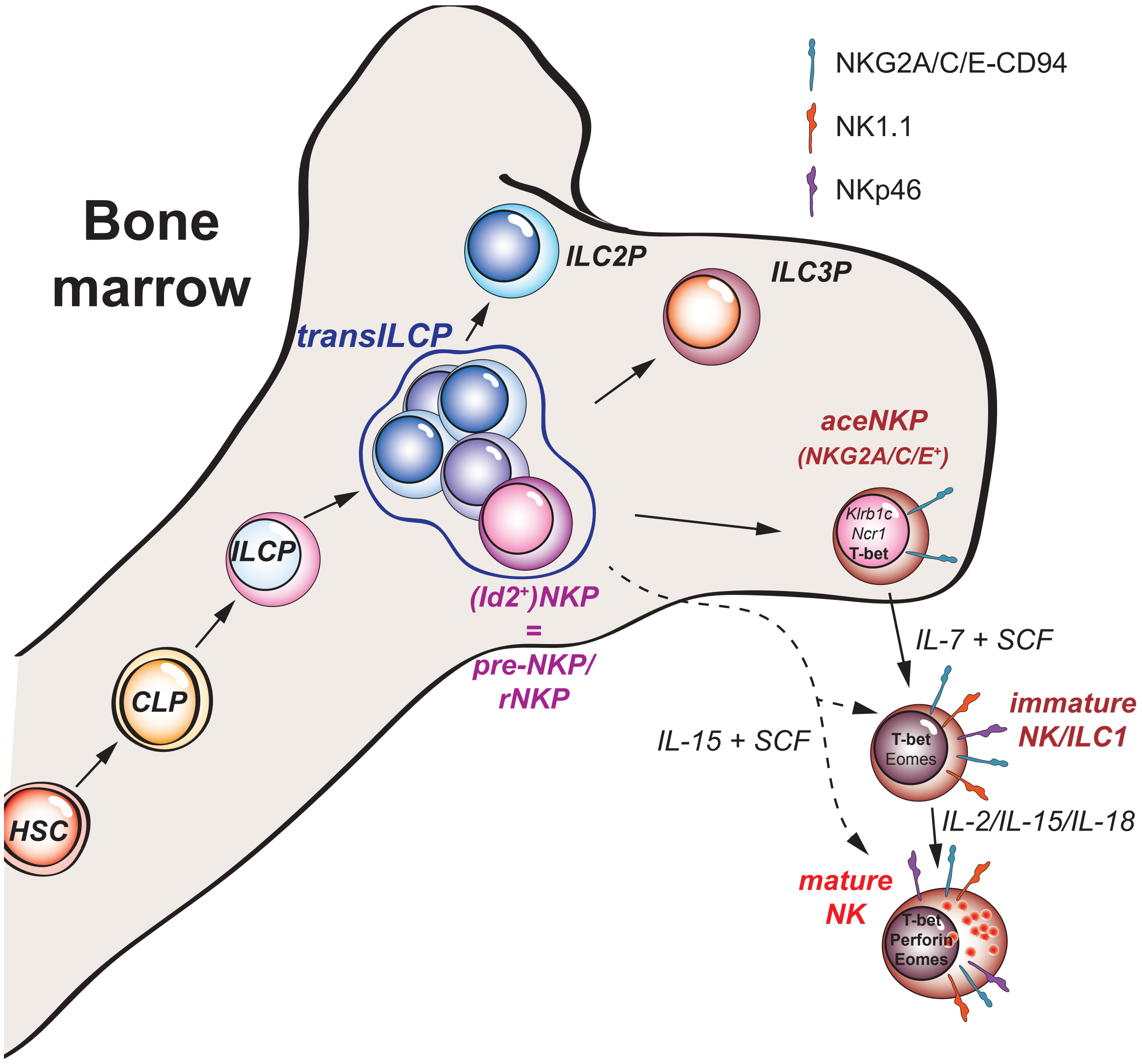
NKG2A/C/E-CD94
NK1.1
NKp46
Bone marrow
HSC
CLP
ILCP
transILCP
ILC2P
ILC3P
(Id2+)NKP = pre-NKP/ rNKP
aceNKP (NKG2A/C/E+)
Klrb1c Ncr1 T-bet
IL-7 + SCF
IL-15 + SCF
immature NK/ILC1
T-bet Eomes
IL-2/IL-15/IL-18
mature NK
T-bet Perforin Eomes

Figure S8. Outline of aceNKP development.

SI References

82. S. Van Gassen *et al.*, FlowSOM: Using self-organizing maps for visualization and interpretation of cytometry data. *Cytometry A* **87**, 636-645 (2015).
83. J. H. Levine *et al.*, Data-Driven Phenotypic Dissection of AML Reveals Progenitor-like Cells that Correlate with Prognosis. *Cell* **162**, 184-197 (2015).
84. J. D. Shields, I. C. Kourtis, A. A. Tomei, J. M. Roberts, M. A. Swartz, Induction of lymphoidlike stroma and immune escape by tumors that express the chemokine CCL21. *Science (New York, N.Y.)* **328**, 749-752 (2010).
